# Important Topics for Fostering Research Integrity by Research Performing and Research Funding Organizations: A Delphi Consensus Study

**DOI:** 10.1007/s11948-021-00322-9

**Published:** 2021-07-09

**Authors:** Krishma Labib, Rea Roje, Lex Bouter, Guy Widdershoven, Natalie Evans, Ana Marušić, Lidwine Mokkink, Joeri Tijdink

**Affiliations:** 1 Department of Ethics, Law and Humanities, Amsterdam UMC, Vrije Universiteit Amsterdam, Amsterdam Public Health Institute, De Boelelaan 1117, 1081HV Amsterdam, The Netherlands; 2 School of Medicine, University of Split, Šoltanska ul. 2, 21000 Split, Croatia; 3 Department of Epidemiology and Data Science, Amsterdam UMC, Vrije Universiteit Amsterdam, Amsterdam Public Health Institute, De Boelelaan 1117, 1081HV Amsterdam, The Netherlands; 4 Department of Philosophy, Vrije Universiteit Amsterdam, De Boelelaan 1105, 1081 HV Amsterdam, The Netherlands

**Keywords:** Research integrity, Research integrity policy, Research performing organizations, Research funding organizations, Institutional policies

## Abstract

To foster research integrity (RI), it is necessary to address the institutional and system-of-science factors that influence researchers’ behavior. Consequently, research performing and research funding organizations (RPOs and RFOs) could develop comprehensive RI policies outlining the concrete steps they will take to foster RI. So far, there is no consensus on which topics are important to address in RI policies. Therefore, we conducted a three round Delphi survey study to explore which RI topics to address in institutional RI policies by seeking consensus from research policy experts and institutional leaders. A total of 68 RPO and 52 RFO experts, representing different disciplines, countries and genders, completed one, two or all rounds of the study. There was consensus among the experts on the importance of 12 RI topics for RPOs and 11 for RFOs. The topics that ranked highest for RPOs concerned education and training, supervision and mentoring, dealing with RI breaches, and supporting a responsible research process (e.g. through quality assurance). The highest ranked RFO topics concerned dealing with breaches of RI, conflicts of interest, and setting expectations on RPOs (e.g. about educating researchers about RI). Together with the research policy experts and institutional leaders, we developed a comprehensive overview of topics important for inclusion in the RI policies of RPOs and RFOs. The topics reflect preference for a preventative approach to RI, coupled with procedures for dealing with RI breaches. RPOs and RFOs should address each of these topics in order to support researchers in conducting responsible research.

**Supplementary Information:**

The online version contains supplementary material available at 10.1007/s11948-021-00322-9.

## Background

Fostering research integrity (RI) is important to ensure trustworthy research (Drenth et al., 2010; Horn, 2013). We see RI as a spectrum of research practices, with serious misconduct (e.g. fabrication, falsification, plagiarism) found on one end of the extremes, responsible research practices (i.e. research conducted according to high professional standards (Boehme et al., 2016)) found on the other end, and questionable research practices (QRPs, e.g. hypothesizing after the results are known) found in between (Steneck, 2006). RI is influenced by multiple factors, including (i) the virtuousness of individual researchers, (ii) the institutional research climate, and (iii) the wider system-of-science (e.g. incentive structures) (All European Academies, 2017; Joynson & Leyser, 2015; Rifai et al., 2019; Titus & Bosch, 2010). To foster RI, it is important to consider each of these factors (All European Academies, 2017; Forsberg et al., 2018; Joynson & Leyser, 2015; Rifai et al., 2019; Titus & Bosch, 2010). While most researchers could be considered well-intentioned, motivated by a desire to improve their understanding of the world, and striving to conduct research with integrity (Joynson & Leyser, 2015), they might be deterred from engaging in responsible research practice when their institutional environment does not support them sufficiently, or when they are faced with perverse incentives, for instance related to the funding system (Joynson & Leyser, 2015; Titus & Bosch, 2010). Currently, many RI initiatives focus on addressing individual researchers’ responsibilities in conducting research responsibly (e.g. by setting requirements on individual researchers) (Zwart & ter Meulen, 2019). However, knowledge on the institutional and system-of-science factors influencing RI is still limited (Richman & Richman, 2012), and further research is needed to understand and tackle these factors (Bruton et al., 2020; Council of the European Union, 2015).

Various stakeholders such as research performing organizations (RPOs) (e.g. universities, independent research institutes, contract research organizations, etc.), research funding organizations (RFOs), journals, national policy makers, and publishers influence the institutional and system-of-science factors of RI (Bouter, 2018; Hermeren et al., 2019). Of these, RPOs and RFOs are particularly interesting, because RPOs have a direct impact on the institutional research climate (VSNU, 2018), while RFOs have a direct impact on elements within the system-of-science (e.g. incentive structures) (Titus & Bosch, 2010). RFOs can also have an indirect impact on the institutional research climate, since they have the means to influence institutional policies of RPOs by setting funding requirements (Tereskerz & Mills, 2012). By addressing the responsibilities of RPOs and RFOs regarding RI, it is possible to tackle some of the institutional and system-of-science factors influencing RI. While existing documents provide RPOs and RFOs with aspirational principles to follow to foster RI (e.g. All European Academies, 2017), there is a lack of concrete guidance available on how to implement these principles in practice (Mejlgaard et al., 2020). An RI policy containing a comprehensive set of concrete infrastructures, trainings, and support systems aimed at fostering RI can provide RPOs and RFOs with the means to apply aspirational RI principles to practice (Bouter, 2020; Lerouge & Hol, 2020). While many institutions globally have begun to implement various initiatives and policies on different aspects of RI (Mejlgaard et al., 2020), they often lack a comprehensive plan that addresses RI systematically. This is why the European Union’s next Horizon Framework program asks institutions receiving funding to state that they have a comprehensive RI plan (Mejlgaard et al., 2020).

The first step to developing a comprehensive RI policy at RPOs and RFOs is to identify which topics to include. For instance, the Bonn-Printeger statement lists several topics that RPOs should address to foster RI, such as providing RI education, improving the organizational research culture, protecting whistle-blowers, etc. (Forsberg et al., 2018). Similarly, the International Funders’ Collaboration ‘Ensuring value in research’ highlights several elements related to RI that RFOs should address, such as research design, and reporting (EViR Funders' Forum, 2020). Although this shows that several RI topics have been identified as important in various national or international documents (Bruton et al., 2020), there is currently no European level consensus among research policy experts and institutional leaders about which topics should be included in the RI policies of RPOs and RFOs. In this study, we used a Delphi survey method to fill this gap. Our first objective was to explore what to address in the institutional RI policies of RPOs and RFOs by seeking consensus from research policy experts and institutional leaders on which RI topics are important for RPOs and RFOs. After achieving this objective, we set an additional second objective: to, with the experts’ input, rank the RI topics in priority to identify which topics should be included first in RI policies. Since RPOs and RFOs likely influence RI in different ways (Tereskerz & Mills, 2012; Titus & Bosch, 2010; VSNU, 2018), we divided the study into two parts, of which Part 1 focused on RPOs and Part 2 focused on RFOs.

## Methods

The key characteristics of a Delphi study include: (1) recruiting an anonymous panel of experts (Diamond et al., 2014), (2) sending multiple rounds of surveys to the panel (Pare et al., 2013), (3) providing feedback to experts in between rounds, based on the results of the previous round (Pare et al., 2013), and (4) seeking out experts’ views on a specific topic (Keeney et al., 2006). As such, Delphi studies use a structured and anonymous data collection process on a purposive sample of experts’ views over several rounds of questionnaires with the purpose of informing decision making (Brady, 2015). Delphi studies do not aim at creating new knowledge, but rather on organizing and structuring existing knowledge based on experts’ views (Powell, 2003). While there are numerous forms of Delphi studies available (Keeney et al., 2001; Powell, 2003), we used the ‘modified’ Delphi approach, starting with a document search before constructing and sending the first questionnaire round to make use of existing literature and minimize time demands on the recruited experts (Brinkman et al., 2018). We used both qualitative and quantitative measures in the Delphi study to ensure that experts had sufficient room to make suggestions and provide comments, and we strongly relied on the qualitative data to interpret the quantitative results. The study was conducted under the guidance of a Delphi expert (LM). The methods have also been described in the preregistered study protocol (https://osf.io/ne85b/) and deviations from study protocol have been added later (https://osf.io/bcjyu/).

### Document search

National and international European RI policy documents were identified to see which RI issues have already been addressed by RPOs’ and RFOs’ policies, using the following search terms on Google: ‘*(research integrity OR research ethics) AND ([*Country or *‘Europe’]) AND (guidelines OR codes of conduct)’*, followed by an exploration of links found on relevant pages. Additionally, using the search terms ‘*(research institution OR university) AND [Country]*’ (RPOs) and ‘*research funding AND [country]’* (RFOs), followed by an exploration of links found on relevant pages, institution-specific policy documents (e.g. standard operating procedures, guidelines, codes, policy statements) were searched from 1 RPO and 1 RFO in each country in the European Research Area (proceeding through countries in alphabetical order). RI issues (e.g. ‘RI education’ and ‘data management facilities’) were extracted from the identified documents. The search for documents ended once saturation was reached for data extraction (i.e. when the same issues kept repeating in subsequent documents and no new issues arising) (Saunders et al., 2018). Based on the issues extracted, and considering overlap and the relationship between issues, two preliminary lists of topics and subtopics were created for 1) RPOs and 2) RFOs. KL was responsible for the document search, extraction of issues, and creation of the preliminary lists of topics. After discussion of the topic lists among all authors, the lists were refined further (e.g. altering phrasing, adding additional topics, etc.).

### Participant selection

The inclusion criteria for study participants (i.e. the ‘experts’) was: people with (1) experience in research policy, and (2) working at RPOs or RFOs. A purposive sampling technique was used to identify experts and consisted of simultaneously approaching personal contacts followed by snowballing, and performing a web search of contacts at RPOs and RFOs across Europe. For the web search of RPO experts, we looked for RI or research policy contacts by browsing the website of three RPOs in each European country; we selected the three RPOs that appeared first in a Google search of ‘(research institution OR university) AND [Country]’. For the RFO experts, we searched the website of at least one RFO in each European country by searching for ‘research funding AND [country]’ on Google. In many countries, only a single RFO could be found; in the case that we were able to identify multiple national RFOs using that search method, we included experts from additional RFOs from the country. We supplemented this search strategy by looking for the contact details of authors of documents we identified in our document search. All experts identified were invited to participate in the study. The experts’ identities remained anonymous to all until study completion, except KL and JT, who were responsible for selection and correspondence.

### Procedures

**Fig. 1:**
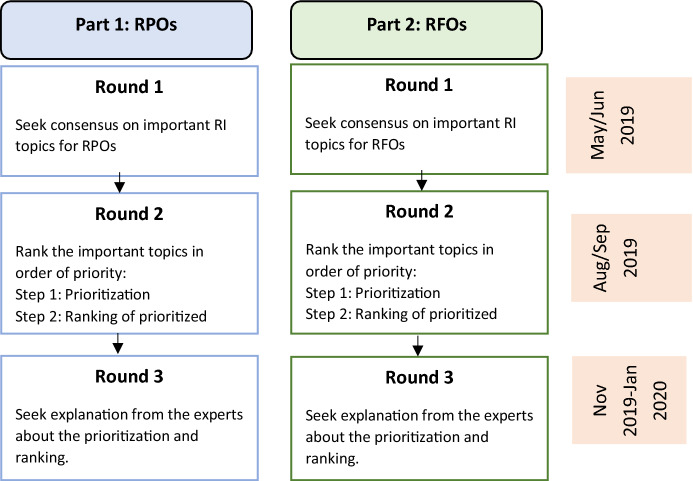
The Delphi procedure

Our study consisted of two parts, each with three online Qualtrics survey rounds, as depicted in Fig. 1. In each survey round, experts were provided with an updated description of each topic and subtopic presented, both in a separate PDF (available at https://osf.io/jc6u2/ for RPOs, and https://osf.io/82dwk/ for RFOs), as well as in popup text included in the survey when the topics or subtopics were mentioned. We refined descriptions each round by incorporating the input from the experts from the previous round.

#### Pilot tests

Before we sent the Round 1 surveys to all experts, we decided to first pilot them with 2 experts from RPOs and 3 experts from RFOs to ensure the clarity of concepts and questions. The pilot experts were personal contacts who met the study inclusion criteria and showed enthusiasm in the study prior to being invited to participate. Based on the feedback of the pilot experts, we made some final adjustments in the survey (e.g. rephrasing some questions) and refined the lists of topics further (e.g. renaming topics). More information on this can be found in Online Resource 1.

#### Round 1

In Round 1, we asked experts to rate each topic in the preliminary list on a 1–5 Likert rating scale, ranging from the topic is ‘not important at all’, to it is ‘absolutely essential’ for institutional efforts in fostering RI. When experts rated a topic 3 or higher, they were also able to rate the subtopics we had identified under that topic, by selecting to ‘Include’ or ‘Do not include’ the subtopic. We randomized the order of the topics, to control for any biases that could occur due to the order of the topic presentation. We used a forced response feature on the survey when asking the experts to rate the topics; we did not force the experts to rate the subtopics. Experts were encouraged to provide arguments for their ratings in open answer options, as well as to suggest new topics/subtopics. The questionnaires of Round 1 can be found on the Open Science Framework (OSF) (RPOs: https://osf.io/w78bj/ and RFOs: https://osf.io/gp5jt/).

#### Round 2

Since consensus on the topics was already reached in Round 1, in Round 2, we asked experts to complete a prioritization and a ranking exercise in two steps. In Step 1, experts were asked to select half the topics that achieved consensus on importance in Round 1, to *prioritize*. In Step 2, they *ranked* the prioritized topics in order of priority. Additionally, experts were asked to rate the subtopics which had not achieved consensus in Round 1, as well as newly proposed subtopics. We encouraged the experts to provide comments on their ratings, and prioritization and ranking choices. The links to the RPO and RFO questionnaires of Round 2 are https://osf.io/wtu6r/ and https://osf.io/5j642/, respectively.

#### Round 3

After analyzing the results of Round 2, we were unsure about whether experts’ prioritization and ranking were motivated by considerations of (1) the feasibility of creating institutional policies on each topic; (2) the impact that topics could have on research practice; (3) the need for RPOs and RFOs to address certain topics; or some other rationale. Therefore, we used a feedback round, which can be used as a member check to increase rigor in Delphi studies (Brady, 2015), as a third exploratory round, in which we asked experts to share their thoughts on what considerations might underlie the ranking of each topic. The RPO questionnaire of Round 3 can be found here: https://osf.io/qmw94/, while the RFO questionnaire can be found here: https://osf.io/48n97/.

### Data Analysis

#### Quantitative

##### Rating of Topics

To analyze the responses of the rating exercise in Round 1, we had originally defined consensus as agreement among 2/3 of the experts (67%) on ratings 3–5 (moderately important, very important, absolutely essential) per topic and ratings of ‘Include’ per subtopic. However, the threshold for the topics did not allow us to see differences since all topics were deemed at least moderately important by > 80% of the experts. Therefore, we retrospectively raised the threshold for consensus to 67% agreement on ratings 4–5 (very important—absolutely essential). To determine whether to include or exclude a topic or subtopic, we considered whether consensus had been achieved. We excluded responses from experts who completed less than 51% of the topic rating questions from the analysis, as they had not completed an assessment of all topics and subtopics, making it difficult to interpret their results.

##### Prioritization and Ranking of Topics

To create a ranked list of topics, we analyzed the prioritization and the ranking exercise results from Round 2 in three phases, A, B and C:

A. We looked at how often each topic was prioritized in Step 1 of the prioritization and ranking exercise.
B. We calculated the total ranking score per topic. To do this, we had to: (1) assign a ranking number per topic per expert, and then (2) per topic, sum the multiple of each ranking number with the number of experts assigned to it. An example of how the ranking scores were calculated can be found in Online Resource 6 (p. 4). We used the following procedure to assign a ranking number per topic per expert. For the prioritized topics we assigned a number of 1 to the topic ranked lowest in Step 2 of the ranking and prioritization exercise by the expert, with each higher ranked topic receiving a number 1 point above that. We assigned a score of -2,5 for each topic not prioritized by the expert in Step 1 of the ranking and prioritization exercise. Since these non-prioritized topics were not ranked relative to each other, we had to use the same ranking score for all the non-prioritized topics per expect; the ranking score of -2,5 that we used was the mean between the values of 0 and -5 that they would have received had they been ranked relative to each other.
C. We looked at the results of phase A to place the most frequently prioritized topics (i.e. those selected by 50% or more of the experts) higher in the final ranked list of topics than those prioritized less frequently. To see in which order to place the most frequently prioritized topics relative to each other, we used the results of phase B; topics with a higher total ranking score were placed higher in the ranked list. We also compared the ranking scores of the less frequently prioritized topics to order them relative to each other.

#### Qualitative

We looked at experts’ arguments for and against the importance of each topic/subtopic. We used the arguments per topic/subtopic to refine the topic/subtopic descriptions and make them more adequate and specific, as well as to improve proposals in the second round (e.g. if after discussion among the authors, we agreed that an argument for excluding a topic was convincing, we proposed to exclude the topic regardless of the quantitative results). Additionally, when making final decisions about the inclusion and exclusion of the topics, we checked the qualitative data from Rounds 1–3 to see whether experts’ views aligned with the quantitative results. In addition to analyzing the qualitative data per topic/subtopic, we noticed some general patterns regarding experts’ views on RI policy across topics. Thematic analysis was used to explore these general considerations (by KL) (Brady, 2015). To check the reliability of the codes, 25% of the data was also analyzed by an independent coder (RR). Discrepancies were discussed by the two coders (KL and RR) to come to agreement; in case of disagreements, a third coder was consulted to reach this agreement (JT). Based on the discussions of the discrepancies, the first coder (KL) rechecked and adjusted the codes of the 75% of the data that were not second coded.

## Results

### Document Search

After searching for national and international policy documents on RI in Europe, 10 documents (i.e. codes of conduct and guidelines) were identified. Additionally, 18 RPO and 14 RFO institution-specific policy documents (e.g. policy statements, guidelines, etc.) were analyzed from which to extract issues. The decision that data saturation was reached was made while extracting issues from RPO and RFO documents from Poland; therefore no documents from subsequent countries (i.e. those following Poland in alphabetical order) were searched for. An overview of these documents can be found in Online Resource 2. In total, 164 issues for RPOs and 64 issues for RFOs were extracted from the documents (by KL). By removal of duplicates (126 RPO issues and 28 RFO issues), combining content, assessing overlap and the relationship between topics, the extracted issues were captured in 13 topics and 34 subtopics for RPOs, and 11 topics and 18 subtopics for RFO (by KL). The topic lists were discussed among the authors to further refine them (e.g. rename topics, regroup topics, remove, and add topics), which resulted in 14 topics and 36 subtopics for RPOs, and 11 topics and 27 subtopics for RFOs (Online Resource 3).

### Results From the Delphi Rounds

The datasets generated from the Delphi rounds can be found on OSF (https://osf.io/3quj6/).

#### Response Rate and Respondent Characteristics

A total of 305 RPO experts and 215 RFO experts were invited to participate in Rounds 1 and 2, while only responders of Round 1 or 2 or both could participate in Round 3. The response rate for the RPO study was 17% (51/305), 18% (53/305) and 52% (35/68) in Rounds 1, 2 and 3, respectively. For the RFO study, the response rate was 18% (39/215), 12% (37/215) and 46% (24/52) in Rounds 1, 2 and 3, respectively. About half the respondents (47% for RPOs and 52% for RFOs) were personal contacts, or contacts found through snowballing. The demographic data showed diversity in the respondents in terms of gender, country, and the disciplinary background of their organization (Online Resource 4). About half of the participants identified themselves as female (44% RPO and 52% RFO experts) and were employed in Northwestern Europe/Scandinavia (46% RPO and 42% RFO experts). The mean number of years of experience in research policy was 14 for both the RPO and RFO experts. A large majority (> 96%) considered themselves at least moderately experienced in RI issues.

### Important Topics for RI Policy

**Fig. 2:**
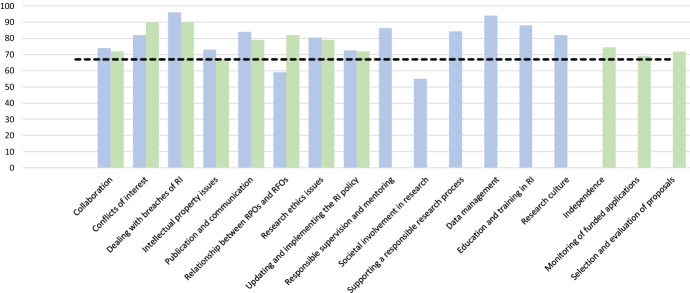
Agreement on importance of RI topics. The x-axis represents the topics that were presented to the experts. The bars in blue indicate the RPO topics, whereas the bars in green indicate the RFO topics. The y-axis represents the percentage of experts who rated each topic 4–5 on importance on the 5 point Likert scale (i.e. very important-absolutely essential). The black dotted line shows the consensus cut-off value of 67% agreement

Of the included 14 topics for RPOs and 11 for RFOs, we achieved consensus on the importance of all but two RPO topics (Fig. 2). Due to a lack of consensus on its importance (59% agreement), and the argument that only RFOs have power to set requirements on RPOs, we excluded the RPO topic ‘Relationship between RPOs and RFOs’. Additionally, we renamed the RFO topic ‘Relationship between RPOs and RFOs’ to ‘Funders’ expectations of RPOs’ to reflect experts’ views that the relationship between RPOs and RFOs is mostly unidirectional. Similarly, we excluded the RPO topic ‘Societal involvement in research’ based on a lack of consensus on its importance (55% agreement) and concerns that the topic is discipline-specific and controversial with one participant stating “If you write a SOP [i.e. a policy document about this topic] you have to be compliant. Is that what you want?” (Round 1, RPO study) and another mentioning that the topic is “not a ‘must’ criteria since not all research might be relevant to this target group [i.e. research stakeholders and policy makers/public authorities]” (Round 1, RPO study). More information about the ratings of the topics and subtopics can be found in Online Resource 5.

### Prioritization of RI Topics

The final ranked lists of RPO and RFO topics, based on the results of Phase C of the analysis of the prioritization and the ranking exercise in Round 2, can be found in Table 1. The topics’ frequency of prioritization (Phase A) and the ranking score per topic (Phase B) can be found in Online Resource 6. The complete ranked list, including the subtopics, can be found in Online Resource 7.

**Table 1:** Ranked list of RI topics

Rank	Topics	Consensus on importance? (% agreement)
*For inclusion in the RI policy of RPOs*		
1	Education and training in RI	Yes (88%)
2	Responsible supervision and mentoring	Yes (86%)
3	Dealing with breaches of RI	Yes (96%)
4	Supporting a responsible research process	Yes (84%)
5	Research ethics issues	Yes (80%)
6	Data management	Yes (94%)
7	Conflicts of interest	Yes (82%)
8	Research culture	Yes (82%)
9	Publication and communication	Yes (84%)
10	Updating and implementing the RI policy	Yes (73%)
11	Intellectual property issues	Yes (73%)
12	Collaborative research among RPOs	Yes (74%)
–	*Relationship between RPOs and RFOs*	*No (59%)*
–	*Societal involvement in research*	*No (55%)*
*For inclusion in the RI policy of RFOs*		
1	Dealing with breaches of RI	Yes (90%)
2	Conflicts of interest	Yes (90%)
3	Funders' expectations of RPOs	Yes (82%)
4	Selection & evaluation of proposals	Yes (72%)
5	Research ethics issues	Yes (79%)
6	Collaboration	Yes (72%)
7	Monitoring of funded applications	Yes (69%)
8	Updating and implementing the RI policy	Yes (72%)
9	Independence	Yes (74%)
10	Publication	Yes (79%)
11	Intellectual property issues	Yes (67%)

### Qualitative Results

Overall, the quantitative results (ratings and rankings) were reflected in experts’ qualitative responses. The qualitative responses from each Delphi round also helped to refine the descriptions of the topics and subtopics presented to the experts in the next round (please see Online Resource 8 to see the evolution of the topic descriptions). These changes were deliberated improvements or additions to the descriptions, rather than major deviations. An example of a definition that changed across rounds was the subtopic ‘Secure data collection, storage, retention, archiving and sharing infrastructure’ falling under the topic ‘Data management’ in the RPO part of the study. Initially we had named this subtopic ‘Secure data storage infrastructure’, but the experts recommended adjusting this name to clarify that the subtopic includes additional stages of the data management process than just storage. Therefore, we adjusted the name and description of the subtopic.

A summary of experts’ comments on the importance of each topic can be found in Online Resource 9, while a summary of the results of Round 3 can be found in Online Resource 10. Furthermore, we noticed that there were patterns in experts’ comments about RI policy that cut across topics and could be insightful for the interpretation of the study. We used thematic analysis to explore these patterns further. Seven overarching themes emerged from experts’ comments (Table 2).

**Table 2:** Themes identified based on qualitative data from the Delphi study

Theme	Description	Example quotes
Views on RI policy	Experts were ambiguous about the notion of RI policy. They identified risks and opportunities of developing RI policy, among which are: *Risks* - Difficult to capture topics in institutional policy documents (feasibility) - Institutional policies might not be effective *Opportunities* - Raising awareness about RI - Building a comprehensive RI system	“One should avoid 'ethics of science in general'. This is a very wide and multifaceted world of issues. Cannot be covered satisfactorily [in research policy]” (Round 1, RPO survey) “[Including policies on] all of this is important, because many organisations…do not have a comprehensive approach, they only have bits and pieces…but it needs to be a system…” (Round 1, RPO survey)
Broadness of definition of RI	Some experts advocated for a broad definition of RI, whereas others encouraged keeping the definition narrow. For instance, there was uncertainty about whether research ethics, human resource management issues (e.g. appointment of researchers) and legal issues (e.g. intellectual property) fall within the scope of RI	“This is important but also necessary not to make RI a container term overloading it with topics… these issues are HR based but have links to RI so in core they should be handled in HR dept. with the exception of a few clear issues” (Round 1, RPO survey) “The narrow definition of research integrity would create an artificially constructed concept which would ignore many of the aspects that constitute integrity (the wholeness of the person, alignment of values and actions etc.).” (Round 2, RPO survey)
Approach to RI	The experts advocated for certain approaches to RI: - Emphasis should lie on prevention, rather than punishment of breaches of RI - Dealing with breaches is complementary to prevention - It is best to focus on the causes, rather than symptoms, of breaches of RI	“I would like to see a preventive approach more than a punitive approach. I think education, information and communication, mentoring, could be an effective approach to create an institutional good RI environment” (Round 3, RFO survey) “It is entirely possible to deal with RI breaches in a way that supports a positive preventative approach. Indeed a calm and open approach to discussing breaches is a hall mark of a good RI culture. No to witch hunts, yes to open discussion.” (Round 3, RPO survey) “The two subjects are not mutual exclusive—prevention (by training, strong ethical research cultures, responsive supervision, etc.) is essential to fostering sound (and trustworthy) science. However, when detrimental practices are detected, institutional (and national) bodies need to be able to handle them in a clear and transparent manner”(Round 3, RPO survey) “Prevention is important, but ways of dealing with RM [i.e. research misconduct] create and maintain the culture of integrity of an institution.” (Round 3, RFO survey) “The main issues relating RI and publication to both authorship and open science relate to the reward structures around current publication practices. It is the reward structures that need to change.” (Round 3, RPO survey)
Differences	Experts identified that the development of RI policy will be influenced by differences in: - Institutions - Countries - Disciplines - Time (i.e. new developments)	“The topic ‘Supporting a responsible research process’ would seem to apply more to research in the biomedical sciences—less so for qualitative research in the social sciences and humanities” (Round 1, RPO survey)
Interrelatedness of topics	Experts mentioned that many of the topics are interrelated, and that to develop a comprehensive RI policy, they should all be addressed Experts mentioned that some topics can be addressed indirectly through other topics	“Prioritizing does not mean neglecting other aspect of the "package". Properly functioning system should take care of all the aspects.” (Round 3, RFO survey) “This [i.e. research culture] is foundational—underpinning all else…” (Round 3, RPO survey) “Research culture is an overarching concept that is influenced by all other issues…” (Round 3, RPO survey)
Autonomy	Experts highlighted that RI policy should not unnecessarily interfere with the autonomy of RFOs, RPOs and researchers	“Many funding agencies [do] NOT check whether the research plan was followed in detail. Quite often unexpected developments force researchers to change their plan to achieve their goal. Funding agencies should not interfere in this process. On the other side: we notice that applicants submit the same proposal in various calls and when the proposal is funded twice, they change the topic of one of the proposals without notifying the funding agency. The latter action is questionable.” (Round 1, RFO survey)
Responsibility	Experts highlighted that research stakeholders have different responsibilities for RI	“Perhaps [publication was ranked low] because publication is difficult to be dealt in the context of RPOs or RFOs but it needs the involvement of science editors and policy makers.” (Round 3, RFO survey)

## Discussion

To our knowledge, this is the first consensus study which has identified what topics are considered important for fostering RI at RPOs and RFOs according to research policy experts and institutional leaders. While some of these topics are already broadly addressed in European codes of conduct on RI (e.g. All European Academies, 2017; VSNU, 2018), many of them have not yet been adequately implemented in the RI policies of RPOs and RFOs (Bouter, 2020; Lerouge & Hol, 2020). The qualitative data shows that, although there is some variation in the broadness of the definition of RI that is accepted by experts in different institutions and countries, experts prefer focusing on a positive, preventative approach to RI as opposed to a punitive approach. This preference is in line with the literature, which indicates an increasing acknowledgement that a positive approach focused on helping researchers engage in responsible research practice is more desirable in fostering RI (Research integrity is much more than misconduct, 2019; Zwart & ter Meulen, 2019). This is not surprising, given that most of the recognized problems with RI qualify as QRPs—a gray zone of research behaviors that in most cases would not be considered as research misconduct (Bouter et al., 2016). An approach to RI solely focused on punishment might not be able to tackle QRPs in organizations successfully (Iorns & Chong, 2014), because it would place too much emphasis on the individual researcher, and not enough on the institutional and system-of-science factors that influence RI (Drenth, 2015; Kumar, 2010).

Despite the preference for a preventative approach, ‘Dealing with breaches of RI’ was prioritized highly for both RPOs and RFOs. The topic was seen as both feasible to address in institutional policies and urgent (i.e. cases of misconduct must be dealt with once they arise). The experts highlighted that prevention and tackling research misconduct can be complementary, since focusing on prevention does not exclude the importance of handling misconduct cases appropriately. Prevention is arguably more likely to be effective when aimed at QRPs, than outright misconduct. Organizations need to foster an environment of openness and learning to tackle minor misbehaviors, while still holding individuals accountable for outright misconduct (Boysen, 2013). Furthermore, if a ‘systems’ approach to tackling misconduct is taken, where not only researchers, but also other stakeholders (including RPOs and RFOs) are held accountable for contributing towards research misconduct, institutional and system-of-science factors can still be addressed (Kumar, 2010). Additionally, as the experts indicated, dealing with research misconduct is necessary to raise awareness about RI and develop a responsible research culture (Stemwedel, 2014).

The experts in this study stressed that fostering a responsible research culture is key to fostering RI. Yet, the topic ‘Research culture’ was not among the highest ranked topics. However, experts mentioned that it overlapped substantially with other higher ranked topics. In fact, the most highly prioritized topics, ‘Education and training in RI’ and ‘Responsible supervision and mentoring’, are both thought to have a direct impact on research culture (e.g. Geller et al., 2010; Kalichman, 2014; Satalkar & Shaw, 2019). It could be that education and supervision are considered concrete ways for affecting research culture, since learning from mistakes is necessary to create a responsible research culture (Boysen, 2013), leading experts to prioritize them instead of the seemingly vaguer topic ‘Research culture’.

Furthermore, the fact that (1)‘Funders’ expectations of RPOs’ (i.e. funders’ requirements) was ranked highly in the RFO part of the study, and (2) the topic ‘Relationship between RPOs and RFOs’ did not achieve consensus on importance in the RPO part of the study, shows that RPOs and RFOs have different roles and responsibilities regarding RI. Since researchers are dependent on the infrastructures and policies of RPOs, RPOs are directly responsible for supporting researchers in RI (Youngblut & Brooten, 2002). RFOs rely on RPOs for many aspects of RI promotion (e.g. the provision of appropriate data management infrastructure, training, etc.) (Tereskerz & Mills, 2012). Therefore, the relationship between RPOs and RFOs is mostly unidirectional, with RFOs imposing requirements and RPOs having to meet them. As such, while it may be of value for RPOs to have policies that address the relationship with RFOs, it might be that such policies would not be impactful enough—due to the unidirectional relationship between RPOs and RFOs—to be vital in the inclusion of their RI policy.

The RPO topic ‘Societal involvement in research’ also did not achieve consensus to be included in RPOs’ RI policies. Experts argued that the topic is too discipline specific and controversial. This suggests that while the topic might be relevant for some types of research (e.g. fields in which public engagement and inclusion is relevant), it may not be broadly applicable to most research. As such, it may be that it is not an important enough topic to recommend to include in the RI policies of RPOs across Europe.

### Strengths and Limitations

Since the study reached out to two heterogenous expert panels, each consisting of more than 50 participants representing different countries (more than 25 countries per panel), genders, and disciplines, we were successful in reaching out to a diverse range of institutional research policy experts—at least from a geopolitical perspective. This helped to obtain consensus at the European level on a comprehensive set of important RI issues for RPOs and RFOs. Since we did not explore the racial and ethnic diversity of the experts, it could be that our findings are dominated by a white European perspective.

While only two of the RPO topics and none of the RFO topics initially presented to the experts were excluded from the final list of topics, the Delphi rounds provided us with valuable inputs which helped us to remove, add, as well as refine topics and subtopics. Additionally, the study enabled us to rank the important RI issues in priority based on the needs and gaps seen by experts in various contexts, allowing for results which are relevant for RPOs and RFOs across disciplines and countries. Although, the ranking exercise was perceived as difficult, as many experts found all the topics to be important, it helped to provide general guidance to RPOs and RFOs on where to start when developing RI policy. Additionally, our findings help to shed light on which approaches to institutional policies on RI experts prefer.

Through the Delphi method, we were able to systematically and democratically engage with the experts (Powell, 2003). This helped to obtain both quantitative and qualitative data from the respondents. Since the experts’ identities remained anonymous to the other experts and researchers (except for KL and JT), we reduced biases that would occur when participants are well acquainted (e.g. higher status stakeholders dominating the discussion) (Powell, 2003). Of course, by focusing on research policy experts and institutional leaders only, we cannot confirm whether the identified topics are also deemed as important by other research stakeholders. However, other studies we have conducted with various research stakeholders (including senior and junior researchers) validate the topics that emerge from this study (Ščepanović et al., 2019; Sørensen et al., 2020).

Since Delphi studies help with structuring existing knowledge, rather than creating new knowledge, our list of topics likely present consensus about known approaches necessary for fostering RI, and might miss out on novel ways that RI can be addressed (e.g. potentially new approaches to reducing perverse incentives). However, given that many of the topics have not yet been implemented widely across institutions in Europe, these topics can still be considered novel and promising to address. For instance, if institutions have policies in place on the subtopic ‘reducing publication pressure and hyper-competition’ (e.g. by rewarding researchers not only on the number of publications but also other outputs and activities), this can help to create a more responsible research culture.

We obtained a sufficient number of responses, but our response rate (12–18%) was lower than reported elsewhere (e.g. 70% in Brinkman et al., 2018; Terwee et al., 2018). This could be because studies with higher response rates base their rates on the number of experts who declare willingness to participate before receiving the study invitation (Boulkedid et al., 2011; De Villiers et al., 2005; Hamilton & Bowers, 2006; Pare et al., 2013). We sent invites to most participants directly, rather than informing them about the study beforehand, which likely explains the lower response rates. This explanation is supported by the fact that two recent Delphi studies using the same means of reporting their response rates had similar figures to ours (Haven et al., 2020; Mokkink et al., 2020). The possibility that the response rate introduces bias into the study is unlikely to be high, given the diversity in the study participants. Since we approached the Delphi study from a primarily qualitative research methodology where the aim was to explore experts’ perceptions and opinions rather than to obtain generalizable knowledge about the mean impression of certain topics, we do not expect the study validity to have been hampered by the response rate (Keeney et al., 2001).

Another methodological concern in this study was the consensus threshold value of 67%, which we set based on the idea that obtaining consensus by 2/3 of the experts would be sufficient to make a well-informed consensus assessment about a topic. However, there are no set standards about how to measure consensus, nor on what threshold value to choose in Delphi studies (von der Gracht, 2012). The threshold value is to some extent arbitrary and serves an instrumental role in helping to explore differences between the importance of items in the Delphi survey. This is why, although we had initially defined consensus as 67% agreement on ratings 3–5, when we saw (based on the results of Round 1) that this consensus definition did not allow us to differentiate between important versus moderately important topics, we modified the consensus definition to 67% agreement on ratings 4–5. Moreover, in addition to relying on the quantitative data, we also examined the qualitative arguments to see whether including or excluding a topic was in line with the experts’ views. If the qualitative data did not support the consensus reached, we brought up the topic/subtopic in the next Delphi round to ask experts to rate the topic/subtopic again.

### Next Steps

While the ranking provides suggestions on which topics to tackle first, RPOs and RFOs will need to address most of the identified topics to build a comprehensive institutional RI policy. However, more empirical work is needed to guide RPOs and RFOs on how to build effective RI policy on the topics identified. Despite our efforts to differentiate between the topics and subtopics by providing experts with clear descriptions of each, some of the topics remain interrelated and connected to each other (e.g. research culture was thought to be both influenced by and underpinning other topics). This is not surprising considering that RI is a complex phenomenon, consisting of multiple stakeholders and factors, and interventions designed to support it are often intertwined (All European Academies, 2017; Bouter, 2018; Joynson & Leyser, 2015; Rifai et al., 2019; Titus & Bosch, 2010). Therefore, it is necessary to explore the relationship and dependencies between the topics further in order to untangle them in future studies. To tackle RI effectively, it will be necessary to address the causes of breaches, rather than only the consequences (National Academies of Sciences Engineering & Medicine, 2017). Furthermore, future research is needed to explore how to prevent the risk that implementing RI policies will introduce unnecessary administrative burdens and a ‘check-box mentality’ towards RI, where RPO and RFO leaders merely address RI topics to show that they are following necessary RI developments. Such research is necessary to ensure that RI policies are instead sensitive to researchers’ needs, and focus on supporting researchers to engage in responsible research practices through a positive and constructive manner. More insight is needed on how country, discipline and institution-specific differences influence topic-specific institutional policies, especially considering that different experts across Europe view RI in narrower or broader terms. For instance, it might be that different types of institutional policies are needed for different disciplines. This will likely also influence the broadness of the definition of RI that should be applied for developing RI policy in a specific organization. Similarly, differences between countries in the availability of and approach to national RI structures and policies (Godecharle et al., 2013; Hermeren et al., 2019) might influence the way that RPOs and RFOs should address each of the topics identified in this study. For example, RPOs and RFOs in countries with no national RI guidelines or structures might have a greater role to play in building RI policies than those in countries with more established structures, as the latter can partially rely on national structures (Godecharle et al., 2013; Hermeren et al., 2019). Additionally, it is important that RPOs’ and RFOs’ RI policies are sensitive to the social, cultural, and historical factors present in the local context (e.g. communication styles) to internalize RI successfully in the institutional culture (Hermeren et al., 2019). Moreover, RPOs and RFOs should work together with the other important stakeholders, including researchers and journals, to jointly produce RI policies that (1) accurately reflect different stakeholders’ responsibilities and needs, (2) sufficiently take into account country, discipline and institution-specific differences, (3) do not unnecessarily interfere with the autonomy of stakeholders, and 4) adequately promote responsible research practices rather than QRPs or misconduct.

## Conclusions

Despite the growing awareness of the importance of RI, there is still little progress on improving the institutional and systemic factors that influence RI. Since RPOs and RFOs have an important role to play here, they should develop, implement and optimize institutional RI policies. This study has made a first step towards changing the landscape by, together with institutional policy experts, exploring which RI topics should be addressed in the RI policies of RPOs and RFOs. Tackling each topic is necessary to effectively support researchers in conducting responsible research.

## Supplementary Information

Below is the link to the electronic supplementary material.

Supplementary file1 (PDF 131 kb)

Supplementary file2 (PDF 416 kb)

Supplementary file3 (PDF 196 kb)

Supplementary file4 (PDF 343 kb)

Supplementary file5 (PDF 407 kb)

Supplementary file6 (PDF 649 kb)

Supplementary file7 (PDF 136 kb)

Supplementary file8 (DOCX 244 kb)

Supplementary file9 (PDF 214 kb)

Supplementary file10 (PDF 299 kb)

Supplementary file11 (PDF 693 kb)

## Data Availability

The datasets generated and analyzed during the current study are available on the Open science framework, https://doi.org/10.17605/OSF.IO/8PZXF. Based on discussions with our privacy officer, we have removed demographic information from the publicly available data set.

## Abbreviations

RI: Research integrity
RPOs: Research performing organizations
RFOs: Research funding organizations
QRPs: Questionable research practices
