# Supplementary material for: Important Topics for Fostering Research Integrity by Research Performing and Research Funding Organizations: A Delphi Consensus Study
Source: Sci Eng Ethics. 2021 Jul 9;27(4):47. doi: 10.1007/s11948-021-00322-9 (PMC8270794; doi:10.1007/s11948-021-00322-9)
Supplement: Supplementary file 1 — Supplementary file1 (PDF 131 kb) [file 11948_2021_322_MOESM1_ESM.pdf]

Appendix 1: Decisions made based on feedback from the pilots in Round 1

After we conducted the pilots for Round 1 of the Delphi survey, we used the experts' feedback on the survey to make some edits in the surveys. The changes that we made can be found below:

RPO study

- We decided to reformulate the following subtopics falling under 'Collaborative research among RPOs' to make them sound more neutral:
  - 'Collaborating with commercial companies' was reformulated to 'Collaboration between private and public RPOs'
  - 'Collaborating with RPOs in low and middle income countries' was reformulated to 'Collaboration between countries with different R&D infrastructures'

Since the reformulation did not alter the meaning of the subtopics, the pilot respondents' ratings and comments on these subtopics could be included in the data analysis.

- The topic 'Communication with lay audience' became a subtopic under the wider topic of 'Societal involvement in research' (which we added after the pilot based on the pilot experts' suggestions). The following additional subtopics were added to the topic 'Societal involvement in research':
  - 'Inclusion of stakeholders in the conduct of research'
  - 'Interaction with public authorities/policy makers'

When we converted the topic 'Communication with lay audience' into a subtopic, we also had to convert the pilot experts' ratings of the topic:

- Ratings 3-5 for the topic were converted into ratings 'Include' for the subtopic
- Ratings 1-2 for the topic were converted into ratings 'Do not include' for the subtopic

We do not have data on pilot experts' responses to the topic 'Societal involvement in research' and the additional two subtopics ('Inclusion of stakeholders in the conduct of research' and 'Interaction with public authorities/policy makers', because the pilot experts were not presented with questions on the topic and subtopics.

RFO study

- We added additional subtopics to the topic 'Selection and evaluation of proposals':
  - 'Research integrity plan'
  - 'Establishing need for research'
  - 'Methodological requirements'.
  - 'Plagiarism'

Since the pilot experts did not have the chance to rate these subtopics, we do not have data from them on these subtopics.

- We moved the subtopic 'Diversity issues' from the topic 'Selection and evaluation of proposals' to the topic 'Research ethics issues'.

The pilot experts suggested that the methodological issues related to diversity issues would be covered under a subtopic on 'Methodological requirements'. Therefore, we decided to move the subtopic to 'Selection and evaluation of proposals' to cover the ethical issues related to diversity in research.

Since the pilot experts rated this subtopic (albeit under a different topic), we included their responses to the subtopic in the analysis.

- We removed the subtopic 'Assessing ethics requirements' from the topic 'Research ethics issues', since the pilot experts mentioned that this topic is redundant (i.e. assessment of general RI requirements of funded project is already included under the topic 'Monitoring').

Since we removed the subtopic, we also excluded the pilot experts' responses to the subtopic from the analysis.

- We made the following changes to the topic 'Independence and academic freedom', to differentiate between interferences that funders themselves could impose on researchers from interferences that external influence could impose on researchers via the funder (e.g. governments or commercial companies).
    - We added the subtopic 'Preventing unjustifiable interference by the funding agency'
    - We reformulated the subtopic 'Preventing unjustifiable interference due to political or intellectual allegiances, or other biases' to 'Preventing unjustifiable interference due to political or other external influences'.
    - We did not alter the phrasing of 'Preventing unjustifiable interference by commercial influences', but we changed its description to reflect that this subtopic is about influences from external commercial influences rather than from the funder itself.

Since we did not make this distinction in the pilot survey, i.e. the subtopics that the expert pilots were presented with (i.e. 'Preventing unjustifiable interference due to political or intellectual allegiances, or other biases' and 'Preventing unjustifiable interference by commercial influences') were explained as coming from the funders directly rather than indirectly from other external influences), we could only include experts' responses to these subtopics under the subtopic 'Preventing unjustifiable interference by the funding agency' in the analysis. We ended up doing this, since there was no differences in the pilot experts' responses to the originally presented subtopics (i.e. if they rated to 'Include' the subtopic 'Preventing unjustifiable interference due to political or intellectual allegiances, or other biases', they did the same for 'Preventing unjustifiable interference by commercial influences').
