# Supplementary material for: Important Topics for Fostering Research Integrity by Research Performing and Research Funding Organizations: A Delphi Consensus Study
Source: Sci Eng Ethics. 2021 Jul 9;27(4):47. doi: 10.1007/s11948-021-00322-9 (PMC8270794; doi:10.1007/s11948-021-00322-9)
Supplement: Supplementary file 2 — Supplementary file2 (PDF 416 kb) [file 11948_2021_322_MOESM2_ESM.pdf]

Appendix 2: Documents identified in the document search

Table 1: List of documents identified in the document search

Organization	Country	RPO/RFO	Document	Issues extracted
National/international policy documents				
VSNU	Netherlands	RPO	Netherlands Code of Conduct for Research Integrity	Training; Supervision; Research culture; Data management; Publication/dissemination; Ethical norms/procedures
EViR	International	RFO	Guiding principles	Research priorities; Research design/conduct/analysis; Regulation and management of research in relation to benefits/risks; Publication/dissemination/open access
Science Europe	European	Both	Research Integrity in the European Policy Landscape	Definition of RI; Promotion of the responsible conduct of research; Dealing with Misconduct; Whistleblower protection; Awareness; Training; Transparency and preparedness; Formal agreements for collaborative research
University of Tartu/ERC	Estonia	RPO	Estonian Code of Conduct on Research Integrity	Guidelines planning of research; Ensuring transparent and fair financing; Ensuring safety in research; Administration of research data; Publishing; RI training & awareness; Dealing with breaches of integrity
TENK	Finland	None	Responsible conduct of research and procedures for handling allegations of misconduct in Finland	Training; Handling allegations of RI misconduct
ÖAWI	Austria	Both	Guidelines on Good Scientific Practice	Data management; Transparency (including avoiding plagiarism); Handling allegations of research misconduct
Multiple national academies	Belgium	None	Code of Ethics for Scientific Research in Belgium	none

UFM	Denmark	RPOs	Danish Code of Conduct for Research Integrity	Research planning and conduct Data management; Publication and communication; Collaborative research; Conflicts of interest; Education, training & supervision; Dealing with breaches of integrity
Various organizations	France	None	National Charter for Research Integrity	none
Irish Universities Association	Ireland	Both	National Policy Statement on Ensuring Research Integrity in Ireland	Research standards; Education; Collaboration for continuous improvement; Definition of misconduct; Procedures for dealing with misconduct; Sanctions
Institution specific policy documents				
University of Vienna	Austria	RPOs	Code of Conduct	Diversity; Conflict management; Conflict of interest; Use of resources; Data management; Financial administration; Compliance; Ombudsman; Ethics committee
Catholic University Leuven	Belgium	RPOs	Information on website	Supervision and mentoring; Publication and authorship; Data management; Image processing; Disclosure of conflict of interest; Training; Defining misconduct; Handling allegations of misconduct
University of Sofia	Bulgaria	RPOs	Information on website	Consultation and advice on ethics; Foster awareness; Handling cases of research misconduct
University of Split	Croatia	RPOs	Information on website	Quality assurance standards; Writing normative documents (guidelines/SOPs); Evaluations; Monitoring
European University of Cyprus	Cyprus	RPOs	Information on website	Dealing with scientific misconduct; Sanctions
University of Copenhagen	Denmark	RPOs	Code of Practice	Providing definitions; RI bodies (e.g. committee/officer/etc.); Reviewing allegations of misconduct; Sanctions
University of Copenhagen	Denmark	RPOs	Policy for Responsible Conduct of Research	Updating RI policy; Setting standards; Rules & procedures for handling research protocols,

				plans/amendments, approval, etc.; Storage and handling of data; Training/education; Authorship advisor'; Collaboration agreements/contracts/information
University of Helsinki	Finland	RPOs	Information on website	Administrative practices in human resources; Financial management; Data protection ; Ethics review; Handling allegations of misconduct
Université Paris-Saclay	France	RPOs	Information on website	RI bodies; Training; Monitoring; Community building
University of Hamburg	Germany	RPOs	Bylaws for Safeguarding Good Scientific Practice and Avoiding Scientific Misconduct at Hamburg University	Data management; Ombudsperson; Investigating misconduct
University of Debrecen	Hungary	RPOs	Rules and regulations for English program students - Faculties of Medicine, Dentistry, Pharmacy and Public Health	Supervision; Ethics Committee ; Handling allegations of misconduct; Independence; Diversity; Appointments and promotions
University College Cork	Ireland	RPOs	Code of research conduct	Academic freedom; RI bodies (RI officer); Definition of research misconduct; Standard of good research practice; RE bodies (RE Committee); Training and qualifications; Research climate; Whistleblowing/settling disputes; Publication & dissemination; Relationship with funders; Handling allegations of research misconduct
University of Latvia	Latvia	RPOs	Regulations for Academic Integrity at the University of Latvia	Research culture; Responsible RI bodies; Dealing with allegations of misconduct
University of Vilnius	Lithuania	RPOs	Information on website	RI/RE education
University of Luxembourg	Luxembourg	RPOs	Information on website	Ethics review; Dealing with allegations of misconduct; RI/RE bodies
University of Malta	Malta	RPOs	Information on website	Research ethics committee
Vrije Universiteit	Netherlands	RPOs	Information on website	Handling integrity complaints; Advising on RI; RI bodies (committee, advisors, etc.)
Kozminski University	Poland	RPOs	Ethical Code of Conduct	Diversity; Conflict of interest; Academic freedom

FWF	Austria	RFOs	Information on website	Handling allegations of misconduct
FWO	Belgium	RFOs	Information on website	none (topics not disclosed; RI policy mentioned)
Croatian Science Foundation	Croatia	RFOs	Guideline on Conflict of Interest	Preventing and dealing with conflict of interest
Lundbeck Foundation	Denmark	RFOs	Code of Conduct	Peer review and impartiality
Estonian Research Council	Estonia	RFOs	Information on website	Open science
Academy of Finland	Finland	RFOs	Information on website	Violations of RI; Gender equality; Open access and open data
French National Research Agency	France	RFOs	Politique en matiere d'ethique et d'integrite scientifique	Project evaluation; Independence; Confidentiality; Selection of projects; Monitoring of projects; Conflict of interest; Expectations RPOs; Handling allegations or RI misconduct
Deutsche Forschungsgemeinschaft	Germany	RFOs	Safeguarding Good Scientific practice	Guidelines for research proposals; Rules for the use of Funds; Reviewers (COI and confidentiality)
Hungarian Academy of Sciences	Hungary	RFOs	Science Ethics Code	Autonomy of scientific research; Obligations of the researcher; Definition of misconduct; RI bodies (ethics committee); Handling allegations of misconduct
Health Research Board	Ireland	RFOs	Information on website	Research with animals; Research culture; Education; Selection of projects; Monitoring of projects; Research priorities
Collegio Carlo Alberto	Italy	RFOs	Information on website	Ethics Committee; Ethics evaluation procedures
FNR	Luxembourg	RFOs	Ethics Charter and Code of Conduct for Research Assessment	Confidentiality; Impartiality; Data management; Conflict of interest
NWO	Netherlands	RFOs	Information on website	Reporting violations; Interventions in cases of violation
National Science Centre	Poland		The Code of the National Science Centre on Research Integrity and Applying for Research Financing	Setting standards; Authorship guidelines; Conflicts of interest; Teaching, training and supervision; Dealing with allegations of misconduct; Sanctions

***Note**: We also examined the Code of Ethics of Ljubljana University, but did not find issues which were not already extracted. Since we saw that the same issues keep repeating themselves across this and the previous 5-10 documents read, we made the decision that saturation was reached, and we stopped the document search and extraction of issues.*

Table 2: Processing the extracted list of RPO RI issues

**Sorting of all extracted issues**		**Include in the first list of topics and subtopics?**	
Total number of extracted issues including duplicates (from Table 1)	164		
**Issues**	**Number of times found in documents identified (in Table 1)**	**Include?**	**Reasons for exclusion**
Education & training	11	yes	
Supervision	4	yes	
Research culture	3	yes	
Data management & protection	6	yes	
Publication	5	yes	
Ethics issues	10	yes	
Research planning & conduct	4	yes	
Definition of RI/misconduct	5	no	This is clear in codes of conduct.
Dealing with research misconduct	15	yes	
Protection of whistleblowers	2	yes	
Awareness	3	no	This can be captured in education and training
Transparency	3	yes	
Financial management	3	no	Only aspects of this relative to promotion & hiring are relevant for RI
Collaborative research	4	yes	
Conflicts of interest	4	yes	
Research standards	3	yes	Include, but rename to 'requirements'
Sanctions	3	yes	
Diversity	3	yes	

Conflict management	2	yes	
Use of resources	1	no	This is too vague
Data management	3	yes	
Compliance	1	no	This can be captured in dealing with breaches of RI
RI bodies	8	yes	
Authorship	2	yes	
Image processing	1	no	This topic is too discipline specific, but falls under quality assurance
Quality assurance	1	yes	
Writing & updating RI policy	2	yes	
Evaluations	1	yes	Included as 'fair evaluations of researchers'
Monitoring	2	no	This is captured dealing with breaches of RI and quality assurance
Reviewing allegations of misconduct	2	yes	Included as 'procedures for investigating allegations of misconduct'
Human resource management	1	no	Unclear how this is related to RI
Community building	1	yes	
Independence	1	no	Topic is less relevant for RPOs compared to RFOs
Appointments & promotions	1	yes	
Academic freedom	2	no	Topic is less relevant for RPOs compared to RFOs
Qualifications	1	yes	Include as 'career building'
Relationship with funders	1	yes	
Advising on RI	1	yes	
Number of duplicates	38		

Total number of issues after removal of duplicates	126		

Table 3: First list of RPO topics and subtopics

First list of topics and subtopics*
**1. Research planning and conduct**<br>a. Research requirements<br>b. Responsible research assessment<br>c. Transparency guidelines<br>**2. Data management:**<br>a. Privacy issues<br>i. GDPR issues<br>ii. Privacy policy<br>b. Secure data storage infrastructure<br>c. FAIR principles<br>**3. Publication and communication**<br>a. Publication guidelines<br>b. Guidelines on open access & open data<br>c. Authorship guidelines & handling disputes<br>d. Reporting guidelines<br>**4. Collaborative research**<br>a. Within/outside the EU<br>b. With RPOs in developing countries<br>c. With commercial companies<br>**5. Conflicts of interest**<br>a. Clarification of what constitutes a conflict of interest<br>b. Reporting of conflicts of interest

6. **Education & Training in RI**
    a. Pre-doctorate RI trainings
    b. Post-doctorate RI trainings
7. **Supervision & Mentoring**
    a. PhD guidelines
    b. Supervisor requirements and guidelines
8. **Valorization issues**
9. **Research culture**
    a. Fair procedures for appointments, promotions and remuneration
        i. Diversity issues
    b. Support for researchers who are leaving academia
    c. Community building
    d. Conflict management – procedures for managing conflicts
    e. Providing clear instructions and guidelines to researchers
        i. RI counselling and advice
10. **Ethics issues**
    a. Research with humans – procedures
    b. Research with animals – procedures
    c. Ethics committees – set up and responsibilities
    d. Ethics reviews
11. **Dealing with breaches of integrity**
    a. Definition of RI and research misconduct
    b. RI bodies
    c. Protection of whistleblowers
    d. Procedures for investigating allegations of misconduct
    e. Sanctions
12. **Updating RI policy**
13. **Relationship with funders**

**This first list of topics and subtopics were drafted solely by KL. The list was later discussed among the other authors to develop a more advanced 'preliminary list of topics and subtopics' which were then presented to the experts in the Delphi rounds (available in Online Resource 3).*

Table 4: Processing the extracted list of RFO RI issues

**Sorting of all extracted issues**		**Include in first preliminary list of topics and subtopics?**	
Total number of extracted issues including duplicates (from Table 1)	64		
**Issues**	**Number of times found in documents identified (in Table 1)**	**Include ?**	**Reasons for exclusion**
Research design, conduct and analysis	2	no	Topic is less relevant for RFOs compared to RPOs
Priorities	2	no	Topic is not relevant for RI
Publication	1	yes	
Definition of RI/misconduct	3	no	This is clear in codes of conduct.
Dealing with research misconduct	7	yes	
Protection of whistleblowers	1	yes	
Awareness	1	no	This is better captured in education and training
Transparency	2	no	Topic is less relevant for RFOs compared to RPOs
Collaborative research	1	yes	
Research standards	2	no	Captured in 'RI requirements' and 'Selection and evaluation of proposals'
Sanctions	2	yes	
Conflicts of interest	5	yes	
Data management	2	no	Topic is less relevant for RFOs compared to RPOs
Impartiality	2	no	This can be captured under 'Conflicts of interest' and 'Independence'
Open science	2	yes	
Gender equality	1	no	Captured under 'Diversity issues'
Project evaluation & selection	3	yes	
Independence	1	yes	
Confidentiality	3	Yes	
Monitoring	2	yes	

Expectations of RPOs	1	no	Captured under 'Relationship with RPOs'
Requirements research proposals	1	yes	Captured in 'RI requirements' and 'Selection and evaluation of proposals'
Requirements for use of funds	1	no	Captured in 'RI requirements' and 'Financial monitoring'
Reviewers	2	no	This issue lacks content
Autonomy of research	1	yes	Captured under 'Independence'
Researcher requirements	1	yes	Captured in 'RI requirements' and 'Selection and evaluation of proposals'
RI bodies	1	yes	
Research with animals	1	no	Too discipline specific
Research culture	1	no	Topic is less relevant for RFOs compared to RPOs
Education & training	2	no	Topic is less relevant for RFOs compared to RPOs
Ethics committee	2	no	Topic is less relevant for RFOs compared to RPOs
Ethics evaluation	1	yes	
Reporting violations of misconduct	1	yes	
Interventions in cases of misconduct	1	yes	
Authorship	1	yes	
Supervision	1	no	Topic is less relevant for RFOs compared to RPOs
Number of duplicates in the extracted issues	28		
Number of issue categories	36		

Table 5: First list of RFO topics and subtopics

First list of topics and subtopics*
**1. Peer review**<br>a. Confidentiality

b. Conflicts of interest
c. Plagiarism check systems

2. Requirements for proposals to receive funds
a. Requirements for researchers to receive funding
b. Diversity requirements

3. Independence & ensuring academic freedom
a. Political influences

4. Monitoring

5. Publication and dissemination
a. Authorship guidelines
b. Open science

6. Ethics issues
a. Ethics requirements
b. Ethics reporting requirements

7. Dealing with breaches of integrity
a. Collaborating with RPOs
b. Definition of RI and research misconduct
c. RI bodies
d. Procedures for handling allegations of misconduct
e. Sanctions

8. Updating policy

9. Intellectual property issues

10. Handling collaborative conflicts
a. between co-applicants
b. between funder and applicant

11. Relationship with RPOs

**This first list of topics and subtopics were drafted solely by KL. The list was later discussed among the other authors to develop a more advanced 'preliminary list of topics and subtopics' which were then presented to the experts in the Delphi rounds (available in Online Resource 3)*
