# Supplementary material for: Important Topics for Fostering Research Integrity by Research Performing and Research Funding Organizations: A Delphi Consensus Study
Source: Sci Eng Ethics. 2021 Jul 9;27(4):47. doi: 10.1007/s11948-021-00322-9 (PMC8270794; doi:10.1007/s11948-021-00322-9)
Supplement: Supplementary file 3 — Supplementary file3 (PDF 196 kb) [file 11948_2021_322_MOESM3_ESM.pdf]

Appendix 3: Preliminary lists of topics and subtopics

Table 1: Preliminary list of RI topics and subtopics for RPOs

**1. Collaborative research among RPOs**
a. Collaborating with RPOs inside/outside the EU
b. Collaborating with RPOs in low and middle income countries
c. Collaborating with commercial research performing companies
**2. Communication with lay audience**
**3. Conflicts of interest**
a. What constitutes a conflict of interest?
b. Handling conflicts of interest
**4. Data management**
a. Data protection and privacy
b. Secure data storage infrastructure
c. FAIR principles
**5. Dealing with breaches of RI**
a. RI bodies in the organization
b. Protection of whistleblowers
c. Protection of the accused
d. Procedures for investigating allegations of misconduct
e. Sanctions
f. Other consequences of research misconduct
**6. Education and training in RI**
a. Pre-doctorate RI training
b. Post-doctorate RI training
c. Training of RI personnel and teachers
d. RI counselling and advice
**7. Intellectual property issues**
**8. Publication and communication with academic audience**
a. Publication statement
b. Authorship
c. Open science
d. The use of reporting guidelines
**9. Relationship with funders regarding RI**
**10. Research culture**
a. Fair procedures for appointments, promotions, and remuneration
b. Career support
c. Culture building
d. Managing competition and publication pressure
e. Conflict management

**11. Research ethics issues**
a. Set-up and tasks of ethics committees
b. Ethics review procedures
c. Diversity issues
**12. Responsible supervision and mentoring**
a. PhD guidelines
b. Supervision requirements and guidelines
c. Supervision by managers/department heads
**13. Supporting a responsible research process**
a. Research requirements
b. Transparency
c. Quality assurance
**14. Updating and implementing the organizational RI policy**

Table 2: Preliminary list of RI topics and subtopics for RFOs

**1. Collaboration and conflicts**
a. Expectations on collaborative research
b. Handing conflicts between grant co-applicants
c. Handling conflicts within the funding agency
d. Handling conflicts between the funder and grant applicant
**2. Conflicts of interest**
a. Conflicts of interest among committee members
b. Conflicts of interest among staff members
c. Conflicts of interest among reviewers
**3. Dealing with breaches of RI**
a. RI bodies in the organization
b. Breaches by funded researchers
c. Breaches by committee members
d. Breaches by staff members
e. Breaches by reviewers
f. Protection of whistleblowers and the accused
**4. Research ethics issues**
a. Research ethics requirements
b. Ethics reporting requirements
c. Assessing ethics requirements
**5. Independence and academic freedom**
a. Preventing unjustifiable interference due to political or intellectual allegiances, or other biases
b. Preventing unjustifiable interference by commercial influences
**6. Intellectual property issues**
**7. Monitoring of funded applications**
a. Financial monitoring
b. Monitoring of the execution of the research grant
c. Monitoring of compliance with RI requirements
**8. Publication and dissemination**
a. Publication requirements
b. Expectations on authorship
c. Open science
**9. Relationship with RPOs regarding RI**
**10. Selection and evaluation of proposals**
a. RI requirements for receiving funding
b. Diversity issues
c. Checking plagiarism
**11. Updating and implementing the organizational RI policy**
