# Supplementary material for: Important Topics for Fostering Research Integrity by Research Performing and Research Funding Organizations: A Delphi Consensus Study
Source: Sci Eng Ethics. 2021 Jul 9;27(4):47. doi: 10.1007/s11948-021-00322-9 (PMC8270794; doi:10.1007/s11948-021-00322-9)
Supplement: Supplementary file 4 — Supplementary file4 (PDF 343 kb) [file 11948_2021_322_MOESM4_ESM.pdf]

Appendix 4: Response rate and respondent characteristics

Table 1: Characteristics of the respondents in the RPO and RFO studies

Characteristics	Number of responses in the RPO study		Number of responses in the RFO study	
	Round 1	All rounds	Round 1	All rounds
**Response rate**				
Round 1				
complete responses	51/305 (17%)	51/305 (17%)	39/215 (18%)	39/215 (18%)
*incomplete responses**	6/305 (2%)	6/305 (2%)	4/215 (2%)	4/215 (2%)
Round 2				
complete responses		51/305 (17%)		36/215 (12%)
incomplete responses		2/305 (1%)		1/215 (0%)
Round 3				
complete responses		34/68 (50%)		23/52 (44%)
incomplete responses		1/68 (2%)		1/52 (2%)
TOTAL (# of experts participating in one, two or all Delphi rounds)		68/305 (23%)		52/215 (25%)
**RPO type**				
University/university hospital	38 (75%)	49 (72%)		
Industry	1 (2%)	1 (1%)		
Intergovernmental organisation	2 (4%)	5 (7%)		
Independent research institute	7 (14%)	8 (12%)		
Other	5 (10%)	5 (7%)		
Missing	0 (0%)	4 (6%)		
**Disciplinary field of organisation**				
Humanities	16 (31%)	26 (38%)	17 (44%)	22 (42%)

Social sciences	23 (45%)	33 (49%)	20 (51%)	26 (50%)
Natural sciences	21 (41%)	30 (44%)	19 (49%)	25 (48%)
Biomedical sciences	28 (55%)	35 (51%)	23 (59%)	30 (58%)
Missing	1 (2%)	4 (6%)	3 (8%)	7 (13%)
**Gender**				
Female	26 (51%)	30 (44%)	21 (54%)	27 (52%)
Male	24 (47%)	32 (47%)	16 (41%)	19 (37%)
Non-binary	0 (0%)	0 (0%)	0 (0%)	0 (0%)
None of the above	0 (0%)	0 (0%)	0 (0%)	0 (0%)
Prefer not to disclose	1 (2%)	2 (3%)	1 (3%)	2 (4%)
Missing	0 (0%)	4 (6%)	1 (3%)	4 (8%)
**Country**				
Outside Europe	3 (6%)	4 (6%)	7 (18%)	10 (19%)
Northwestern Europe & Scandinavia	26 (51%)	31 (46%)	18 (46%)	22 (42%)
Southwestern Europe	9 (18%)	10 (15%)	2 (5%)	2 (4%)
Northeastern Europe	2 (4%)	5 (7%)	2 (5%)	3 (0%)
Southeastern Europe	4 (8%)	5 (7%)	5 (13%)	6 (11%)
Central Europe	5 (10%)	7 (10%)	3 (8%)	4 (8%)
Missing	2 (4%)	6 (9%)	2 (5%)	5 (10%)
TOTAL number of countries	24	28	24	26
**Research policy experience**				
Mean number of years	13,9	14,3	13,4	13,8
Maximum number of years	40	50	60	60
Minimum number of years	1	1	2	2
Interquartile range	14	13	12.5	12.5
Missing	1	5	2	5

**Degree**				
PhD/Doctorate	40 (78%)	50 (74%)	28 (72%)	35 (67%)
Master	10 (20%)	12 (18%)	10 (26%)	13 (25%)
Bachelor	1 (2%)	2 (3%)	0 (0%)	0 (0%)
Missing	0 (0%)	4 (6%)	1 (3%)	4 (8%)
**RI experience**				
Not experienced at all	1 (2%)	1 (1%)	0 (0%)	0 (0%)
Slightly experienced	2 (4%)	2 (3%)	1 (3%)	1 (2%)
Moderately experienced	22 (43%)	25 (37%)	15 (38%)	21 (40%)
Very experienced	21 (41%)	28 (41%)	12 (31%)	13 (25%)
Extremely experienced	5 (10%)	8 (12%)	10 (26%)	13 (25%)
Missing	0 (0%)	4 (6%)	1 (3%)	4 (8%)

The table shows the response rate and demographic characteristics of the respondents. Some experts participated in all rounds, while others only completed one or two rounds of the Delphi. That is why the TOTALs in the response rate are not a simple sum of the number of respondents in Rounds 1, 2 and 3. For the items on 'Type of RPO' that the experts worked in, 'Disciplinary field of the organisation', and 'Gender', experts could indicate multiple options. For the item 'Country', experts had to state the country that they mainly worked in. The categories of countries seen in the table were grouped as follows: ***Central Europe*** *included Austria, Slovakia, Czech Republic and Hungary;* ***Northwestern Europe and Scandinavia*** *included Ireland, UK, Netherlands, France, Belgium, Luxembourg, Germany, Switzerland, Denmark, Norway, Finland and Sweden****; Southwestern Europe*** *included Portugal, Spain and Italy;* ***Northeastern Europe*** *included Estonia, Latvia, Lithuania, Ukraine, Belarus, Poland and Russia;* ***Southeastern Europe*** *included Romania, Bulgaria, Greece, Moldova, Serbia, Croatia, Slovenia, Bosnia & Herzegovina, Albania and Macedonia. For the item 'Research policy experience', experts had to indicate how many years they had been involved in research policy, while for the item 'Degree' they had to indicate the highest degree earned. Finally, experts were asked to declare how experienced they are in RI ranging from 'Not experienced at all' to 'Extremely experienced'. Experts were provided with the following definition of RI in the first Delphi survey: 'conducting research according to high professional, ethical and methodological standards'. However, experts were not provided with a definition of RI expertise.*The incomplete responses from Round 1 were excluded from any analyses, since less than 50% of the survey had been completed by these respondents. The incomplete responses from Rounds 2 and 3 were included in the analysis. Those who had incomplete responses in Round 1 and did not participate in Round 2 were not invited to participate in Round 3.*

Table 2: The number of respondents who were personal contacts

	RPOs	RFOs
Total number of invitees	305	215
Number of invitees who are personal contacts*	88 (29%)	64 (30%)
Number of invitees found through web-search	217 (71%)	151 (70)%
Total number of respondents (Round 1 and/or 2, 3)	68	52
Number of respondents who are personal contacts*	32 (47%)	27 (52%)
Number of respondents found through web-search	36 (53%)	25 (48%)

**'Personal contacts' here includes both direct personal contacts of the authors, as well as contacts of contacts found through snowballing.*

Table 3: The number participants in each Delphi round

**Number of experts**	**Participants**	
	**RPO study**	**RFO study**
Number of experts per Delphi round		
TOTAL number of experts (R1 and/or R2 and/or R3)	68	52
*Number of experts in R1**	51/68 (75%)	39/52 (75%)
Number of experts in R2	53/68 (78%)	37/52 (71%)
Number of experts in R3	35/68 (51%)	24/52 (46%)
Number of experts in multiple rounds		
Number of exerts in R1 and R2 and R3	20/68 (29%)	17/52 (33%)
Number of experts in R1 and R2	36/68 (53%)	24/52 (46%)
Number of experts in R1 and R3	23/68 (34%)	19/52 (37%)
Number of experts in R2 and R3	32/68 (47%)	22/52 (42%)

**The denominator in this table refers to the total number of experts participating in the Delphi study (R1 and/or R2 and/or R3), rather than the total number of experts invited to the study.*

Table 4: Characteristics of participants participating in one Delphi round vs. multiple rounds

Characteristic	Number of experts			
	RPO study		RFO study	
	Participated in 1 round	Participated in 2 or more rounds	Participated in 1 round	Participatedd in 2 or more rounds
**Experts**				
Number of participants	17 (25%)	51 (75%)	20 (38%)	32 (62%)
**RPO type**				
University/university hospital	14 (82%)	35 (69%)		
Industry	0 (0%)	1 (2%)		
Intergovernmental organization	0 (0%)	5 (10%)		
Independent research institute	1 (6%)	7 (14%)		
Other	0 (0%)	5 (10%)		
Missing	2 (12%)	2 (4%)		
**Disciplinary field of organization**				
Humanities	6 (35%)	20 (39%)	9 (45%)	13 (41%)
Social sciences	8 (47%)	25 (49%)	10 (50%)	16 (50%)
Natural sciences	9 (53%)	21 (41%)	10 (50%)	15 (47%)
Biomedical sciences	9 (53%)	26 (51%)	11 (55%)	19 (59%)
Missing	2 (12%)	2 (4%)	4 (20%)	3 (9%)
**Gender**				
Female	7 (41%)	23 (45%)	11 (55%)	16 (50%)

Male	8 (47%)	24 (47%)	5 (25%)	14 (44%)
Non-binary	0 (0%)	0 (0%)	0 (00%)	0 (0%)
None of the above	0 (0%)	0 (0%)	0 (00%)	0 (0%)
Prefer not to disclose	0 (0%)	2 (4%)	1 (05%)	1 (3%)
Missing	2 (12%)	2 (4%)	3 (15%)	1 (3%)
**Country**				
Outside Europe	1 (6%)	3 (6%)	2 (10%)	8 (25%)
Northwestern Europe & Scandinavia	10 (59%)	21 (41%)	8 (40%)	14 (44%)
Southwestern Europe	2 (12%)	8 (16%)	2 (10%)	0 (0%)
Northeastern Europe	1 (6%)	4 (8%)	1 (5%)	2 (6%)
Southeastern Europe	0 (0%)	5 (10%)	2 (10%)	4 (13%)
Central Europe	1 (6%)	6 (12%)	1 (5%)	3 (9%)
Missing	2 (12%)	4 (8%)	4 (20%)	1 (3%)
TOTAL number of countries	12	16	14	12
**Research policy experience**				
Mean number of years	18	13	12,5	14,5
Maximum number of years	40	50	23	60
Minimum number of years	6	1	2	3
Interquartile range	13	12	9	13
Missing	2	3	3	2
**Degree**				
PhD/Doctorate	13 (76%)	37 (73%)	13 (65%)	22 (69%)
Master	2 (12%)	10 (20%)	4 (20%)	9 (28%)
Bachelor	0 (0%)	2 (4%)	0 (0%)	0 (0%)
Missing	2 (12%)	2 (4%)	2 (10%)	2 (6%)
**RI experience**				

Not experienced at all	0 (0%)	1 (2%)	0 (0%)	0 (0%)
Slightly experienced	1 (6%)	1 (2%)	0 (0%)	1 (3%)
Moderately experienced	4 (24%)	21 (41%)	7 (35%)	14 (44%)
Very experienced	6 (35%)	22 (43%)	6 (30%)	7 (22%)
Extremely experienced	4 (24%)	4 (8%)	4 (20%)	9 (28%)
Missing	2 (12%)	2 (4%)	3 (15%)	1 (3%)
