# Supplementary material for: Important Topics for Fostering Research Integrity by Research Performing and Research Funding Organizations: A Delphi Consensus Study
Source: Sci Eng Ethics. 2021 Jul 9;27(4):47. doi: 10.1007/s11948-021-00322-9 (PMC8270794; doi:10.1007/s11948-021-00322-9)
Supplement: Supplementary file 5 — Supplementary file5 (PDF 407 kb) [file 11948_2021_322_MOESM5_ESM.pdf]

Appendix 5: Rating of topics and subtopics on importance

Table 1: The rating of topics and subtopics – RPO study

Topics & subtopics	Agreement on importance	
	Round 1	Round 2
**1. Collaborative research among RPOs**	**38/51 (75%)**	
a. within/outside the EU	42/49 (86%)	
b. between countries with different R&D infrastructures	39/49 (80%)	
c. between public and private RPOs	45/51 (88%)	
**2. Conflicts of interest***	**42/51 (82%)**	
a. In peer review		47/49 (96%)
b. In the conduct of research		40/49 (82%)
c. In appointments and promotions		40/49 (82%)
d. In research evaluations		47/49 (96%)
e. In consultancy		37/49 (76%)
**3. Data management**	**48/51 (94%)**	
a. Data protection and privacy	46/51 (90%)	
b. Secure data storage infrastructure	43/51 (84%)	
c. FAIR principles	46/50 (92%)	
**4. Dealing with breaches of RI**	**49/51 (96%)**	
a. RI bodies	45/51 (88%)	
b. Protection of whistleblowers	44/49 (90%)	
c. Protection of those accused of research misconduct	47/49 (96%)	
d. Procedures for investigating allegations of misconduct	48/51 (94%)	
e. Sanctions	36/50 (72%)	36/49 (73%)
f. Other actions in case of misconduct	43/49 (88%)	
**5. Education and training in RI**	**45/51 (88%)**	
a. Pre-doctorate RI trainings	45/51 (88%)	
b. Post-doctorate RI trainings	44/50 (88%)	
c. Training of RI personnel and teachers	48/51 (94%)	

d. RI counselling and advice	45/51 (88%)	
**6. Intellectual property issues**	**37/51 (73%)**	
a. Policies ensuring compliance with IP regulations		38/49 (78%)
b. Interaction of IP and open science requirements		40/49 (82%)
c. Legal counselling		25/49 (51%)
**7. Publication and communication**	**43/51 (84%)**	
a. Publication statement	39/47 (83%)	
b. Authorship	48/50 (96%)	
c. Open science	41/50 (82%)	
d. The use of reporting guidelines	39/48 (81%)	
e. Peer review		46/49 (94%)
f. Predatory publishing		43/49 (88%)
g. Communicating with the public		40/49 (82%)
**8. Relationship between RPOs and RFOs**	**30/51 (59%)**	
**9. Research culture**	**42/51 (82%)**	
a. Fair procedures for appointments, promotions and remuneration	41/49 (84%)	
b. Adequate education and skills training**	34/48 (71%)	41/49 (84%)
c. Culture building	39/51 (76%)	
d. Managing competition and publication pressure	39/50 (78%)	
e. Conflict management	44/51 (86%)	
f. Diversity issues***		37/49 (76%)
**10. Research ethics issues**	**41/51 (80%)**	
a. Set-up and tasks of ethics committees	39/49 (80%)	
b. Ethics review procedures	44/49 (90%)	
c. Diversity issues***	33/46 (72%)	
**11. Responsible supervision and mentoring**	**44/51 (86%)**	
a. PhD guidelines	42/50 (84%)	
b. Supervision requirements and guidelines	46/51 (90%)	
c. Building and leading an effective team	36/50 (72%)	38/49 (78%)

**12. Societal involvement in research**	**27/49 (55%)**	
a. Communicating with lay audience/stakeholders	36/50 (72%)	
b. Inclusion of stakeholders in the conduct of research	26/48 (54%)	
c. Interaction with public authorities/policies makers	32/48 (67%)	
**13. Supporting a responsible research process**	**43/51 (84%)**	
a. Research requirements	44/51 (86%)	
b. Transparency	42/49 (86%)	
c. Quality assurance	38/48 (79%)	
**14. Updating and implementing the RI policy**	**37/51 (73%)**	

'Agreement on importance' refers to the number and percentage of participants who 1) rated topics as 4-5 on the 5 point Liker scale (very important-absolutely essential), and 2) voted to 'Include' (as apposed to 'Exclude) subtopics. In Round 1, experts had to rate the preliminary topics and subtopics. Please note that experts were explicitly given the opportunity to rate a subtopic if they had rated the topic that the subtopic belonged under as 3-5 on the 5 point Likert scale (of moderate importance – absolutely essential). However, since experts who rated the overall topic as 1-2 (not important – of minimal importance) were also presented with the subtopic information when asked to rate the overall topic, we assumed that they would also exclude the subtopics falling under the overall topic and we counted their response for the subtopics as 'Exclude'. The total number of responses for the topics and subtopics might differ since the questions on the topic ratings had a forced response feature, whereas the questions on the subtopic ratings did not.

Subtopics for which there is no data under the column Round 1 were not yet presented to experts in Round 1 (i.e. these were new subtopics that emerged from the comments of the respondents in the first round and were presented to the experts in Round 2). In Round 2, experts had to rank reformulated topics and subtopics; topics and subtopics which did not achieve consensus in Round 1; topics and subtopics which the authors needed further input on based on the qualitative results of Round 1; and new topics and subtopics. We had already reached consensus in Round 1 on whether to include the subtopics for which there is no data under the column 'Round 2'; these subtopics were, therefore, not presented to the experts again in Round 2.

**In Round 1, two other subtopics were included under this topic. Although we achieved consensus to include them, we chose to replace them with the subtopics in the table. For an explanation of this decision, please see https://osf.io/qa9q5/.*

***In Round 1, this subtopic was called 'Career support'.*

****In Round 1, 'Diversity issues' was included under Research ethics issues, but we proposed to move it to 'Research culture' in Round 2.*

*****In Round 1, this subtopic was called 'Supervision by managers/department heads'.*

Table 4: The rating of topics and subtopics – RFO study

**Topic**	**Agreement on importance**	
	**Round 1**	**Round 2**
**1. Collaboration***	**28/39 (72%)**	
a. Expectations on collaborative research	32/38 (84%)	
b. Handling conflicts between grant co-applicants	28/39 (72%)	27/36 (75%)**
c. Handling RI conflicts within the funding agency	24/38 (63%)	
d. Handling RI conflicts between the funder and grant applicant	29/39 (74%)	
e. Research that is co-financed by multiple funders		34/36 (94%)
**2. Conflicts of interest**	**35/39 (90%)**	
a. Among review committee members	36/39 (92%)	
b. Among reviewers	35/39 (90%)	
c. Among staff members	31/38 (82%)	
**3. Dealing with breaches of RI**	**35/39 (90%)**	
a. RI bodies	30/38 (79%)	
b. Breaches by funded researchers	36/39 (92%)	
c. Breaches by review committee members	36/39 (92%)	
d. Breaches by reviewers	37/39 (95%)	
e. Breaches by staff members	32/38 (84%)	
f. Protection of whistleblowers and those accused of research misconduct	31/39 (79%)	
**4. Funders' expectations of RPOs**	**32/39 (82%)**	
a. Codes of Conduct		31/36 (86%)
b. Assessment of researchers		27/36 (75%)
c. Education and training for RI		30/36 (83%)
d. Processes for investigating allegations of research misconduct		35/36 (97%)
**5. Independence**	**29/39 (74%)**	
a. Preventing unjustifiable interference by the funding agency	32/38 (84%)	
b. Preventing unjustifiable interference by political/other external influences	28/35 (80%)	
c. Preventing unjustifiable interference by commercial influences	30/35 (86%)	

d. What counts as an unjustifiable interference?		30/36 (83%)
**6. Intellectual property issues**	**26/39 (67%)**	
**7. Monitoring of funded applications**	**27/39 (69%)**	
a. Financial monitoring	24/36 (67%)	
b. Monitoring of the execution of the research grant	29/37 (78%)	
c. Monitoring of compliance with RI requirements	30/36 (83%)	24/36 (67%)
**8. Publication**	**31/39 (79%)**	
a. Publication requirements	33/36 (92%)	
b. Expectations on authorship	34/38 (89%)	
c. Open science	33/38 (87%)	
**9. Research ethics issues**	**31/39 (79%)**	
a. Research ethics requirements	33/38 (87%)	
b. Diversity issues***	25/38 (66%)	11/36 (31%)
c. Ethics reporting requirements	30/37 (81%)	
**10. Selection and evaluation of proposals**	**28/39 (72%)**	
a. RI plan	29/39 (74%)	
b. Establishing need for research	20/34 (59%)	23/36 (64%)
c. Methodological requirements	28/35 (80%)	
d. Plagiarism	31/39 (79%)	
e. Diversity issues***		19/36 (53%)
**11. Updating and implementing the RI policy**	**28/39 (72%)**	

'Agreement on importance' refers to the number and percentage of participants who 1) rated topics as 4-5 on the 5 point Liker scale (very important-absolutely essential), and 2) voted to 'Include' (as apposed to 'Exclude) subtopics. In Round 1, experts had to rate the preliminary topics and subtopics. Please note that experts were explicitly given the opportunity to rate a subtopic if they had rated the topic that the subtopic belonged under as 3-5 on the 5 point Likert scale (of moderate importance – absolutely essential). However, since experts who rated the overall topic as 1-2 (not important – of minimal importance) were also presented with the subtopic information when asked to rate the overall topic, we assumed that they would also exclude the subtopics falling under the overall topic and we counted their response for the subtopics as 'Exclude'. The total number of responses for the topics and subtopics might differ since the questions on the topic ratings had a forced response feature, whereas the questions on the subtopic ratings did not.

Subtopics for which there is no data under the column Round 1 were not yet presented to experts in Round 1 (i.e. these were new subtopics that emerged from the comments of the respondents in the first round and were presented to the experts in Round 2). In Round 2, experts had to rank reformulated topics and subtopics; topics and subtopics which did not achieve consensus in Round 1; topics and subtopics which the authors needed further input on based on the qualitative results of Round 1; and new topics and subtopics. We had already reached consensus in Round 1 on whether to include the subtopics for which there is no data under the column 'Round 2'; these subtopics were, therefore, not presented to the experts again in Round 2.

**In Round 1, this topic was named 'Collaboration and conflicts', but since we had to exclude all the subtopics related to conflicts (due to the results in Round 1), we renamed the topic to 'Collaboration.*

***We proposed to exclude this subtopic in Round 2, so the agreement is for the exclusion of the subtopic.*

****In Round 1, 'Diversity issues' was placed under Research ethics issues. In Round 2, experts could rate whether to include 'Diversity issues' under the topic 'Selection and evaluation of proposals' (in line with our proposals), under 'Research ethics issues', or to exclude it from the toolbox.*
