# Supplementary material for: Important Topics for Fostering Research Integrity by Research Performing and Research Funding Organizations: A Delphi Consensus Study
Source: Sci Eng Ethics. 2021 Jul 9;27(4):47. doi: 10.1007/s11948-021-00322-9 (PMC8270794; doi:10.1007/s11948-021-00322-9)
Supplement: Supplementary file 6 — Supplementary file6 (PDF 649 kb) [file 11948_2021_322_MOESM6_ESM.pdf]

Appendix 6: Results of the prioritization and ranking exercise

1. Prioritization and ranking of the RPO topics

Figure 1 shows how often each RI topic was prioritized by experts in Step 1 of the prioritization and ranking exercise:

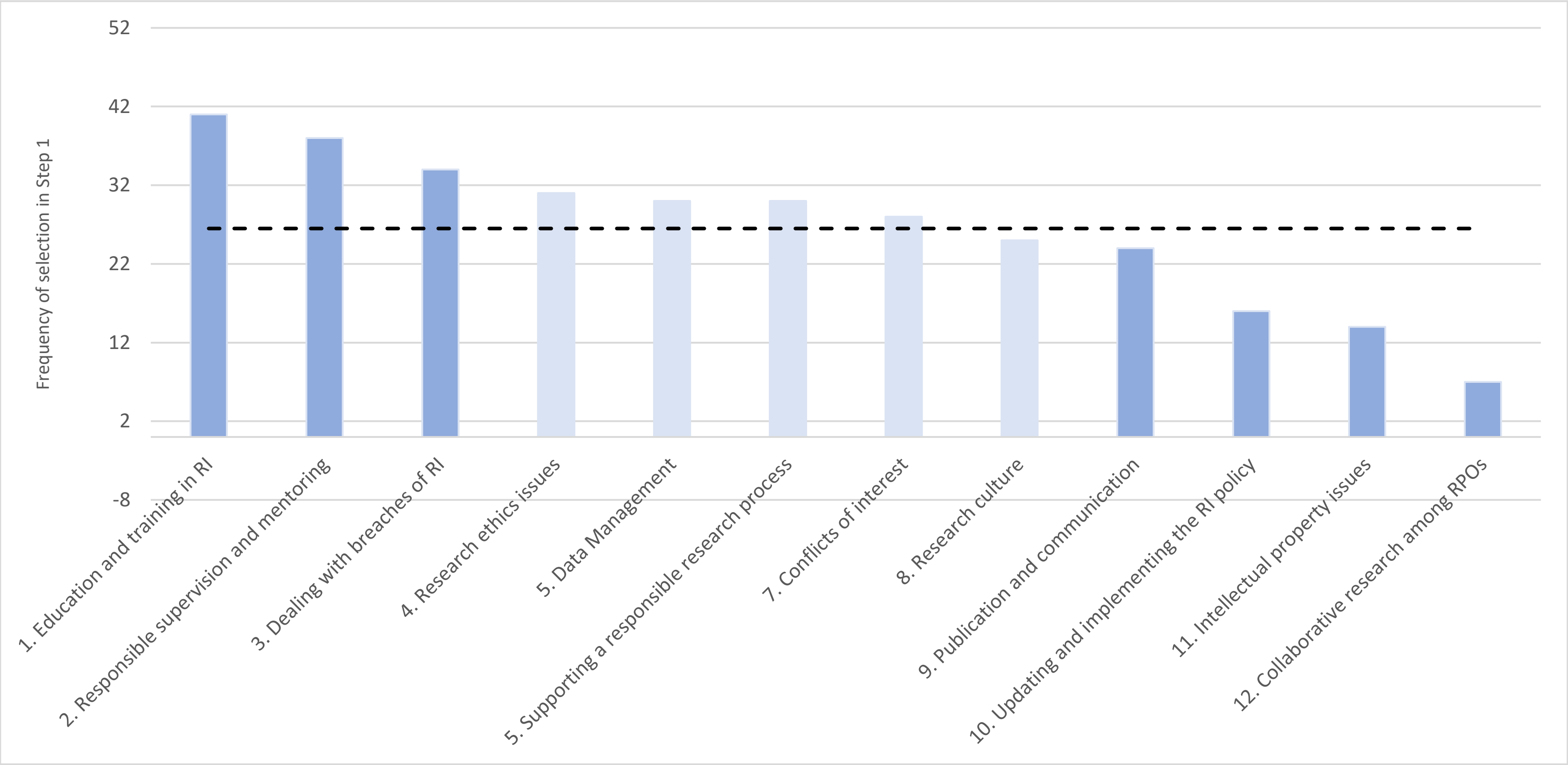

Figure 1 ***How often each topic was prioritized***. *53 experts took part in this exercise, so each topic could be selected a maximum of 53 times. The black dotted line represents 50% of respondents. The bars that are in dark blue represent the topics that received the same ordering in Steps 1 and 2 of the prioritization and ranking exercise.*

Figure 2 shows the total ranking score of each topic.

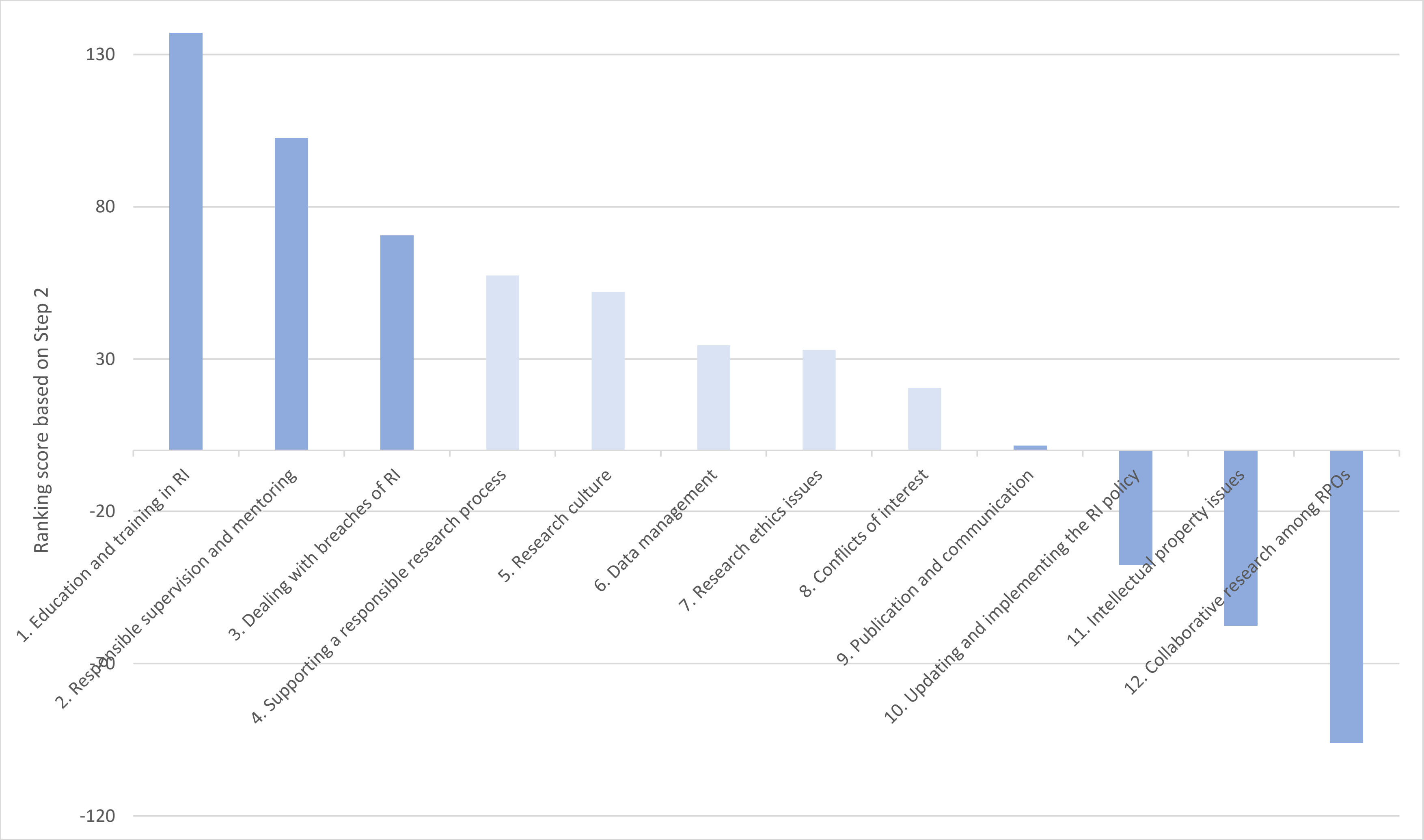

Figure 2 ***The total ranking score of each topic.*** *Topics with a higher ranking score received a higher ranking place. The bars that are in dark blue represent the topics that received the same ordering in Steps 1 and 2 of the prioritization and ranking exercise.*

Figure 3 is an example that illustrates how the total ranking score was calculated for the topic 'Research culture' (Phase B of the analysis of the prioritization and ranking exercise). Table 1 shows an overview of the number of experts who were assigned each ranking score for each topic.

- 28 experts did not prioritize 'Research culture' *[score -2.5]*

Among those who prioritized 'Research culture':

- 3 experts ranked it last *[score 1]*
- 1 expert ranked it second last *[score 2]*
- 2 experts ranked it third last *[score 3]*
- 1 expert ranked it third highest *[score 4]*
- 1 expert ranked it second highest *[score 5]*
- 17 experts ranked it highest *[score 6]*

Total ranking score = (28*-2.5)+(3*1)+(1*2)+(2*3)+(1*4)+(1*5)+(17*6) = **52**

Figure 3 ***Example:*** *Calculating the total ranking score for the topic 'Research culture'*.

Table 1: Ranking scores per topic

Topic	Ranking score							Total ranking score
	**6**	**5**	**4**	**3**	**2**	**1**	**-2.5**	
**Education and training in RI**	9	9	9	7	4	3	12	137
**Responsible supervision and mentoring**	2	10	10	7	8	1	15	102.5
**Dealing with breaches of RI**	4	7	8	4	4	7	19	70.5
**Supporting a responsible research process**	7	5	5	5	5	3	23	57.5
**Research Culture**	17	1	1	2	1	3	28	52
**Data Management**	1	7	4	7	3	8	23	34.5
**Research ethics issues**	5	1	3	5	9	8	22	33
**Conflicts of interest**	3	3	3	5	9	5	25	20.5
**Publication and communication**	3	4	2	4	5	6	29	1.5
**Updating and implementing the RI policy**	1	3	4	4	2	2	37	-37.5
**Intellectual property issues**	0	3	3	1	3	4	39	-57.5
**Collaborative research among RPOs**	1	0	1	2	0	3	46	-96

This table represents the number of experts (text not in bold) who were assigned each ranking score per topic in Phase B of the analysis of the prioritization and ranking exercise.

2. Prioritization and ranking of the RFO topics

Figure 4 shows how often each RI topic was selected to be prioritized by experts in Step 1 of the prioritization and ranking exercise:

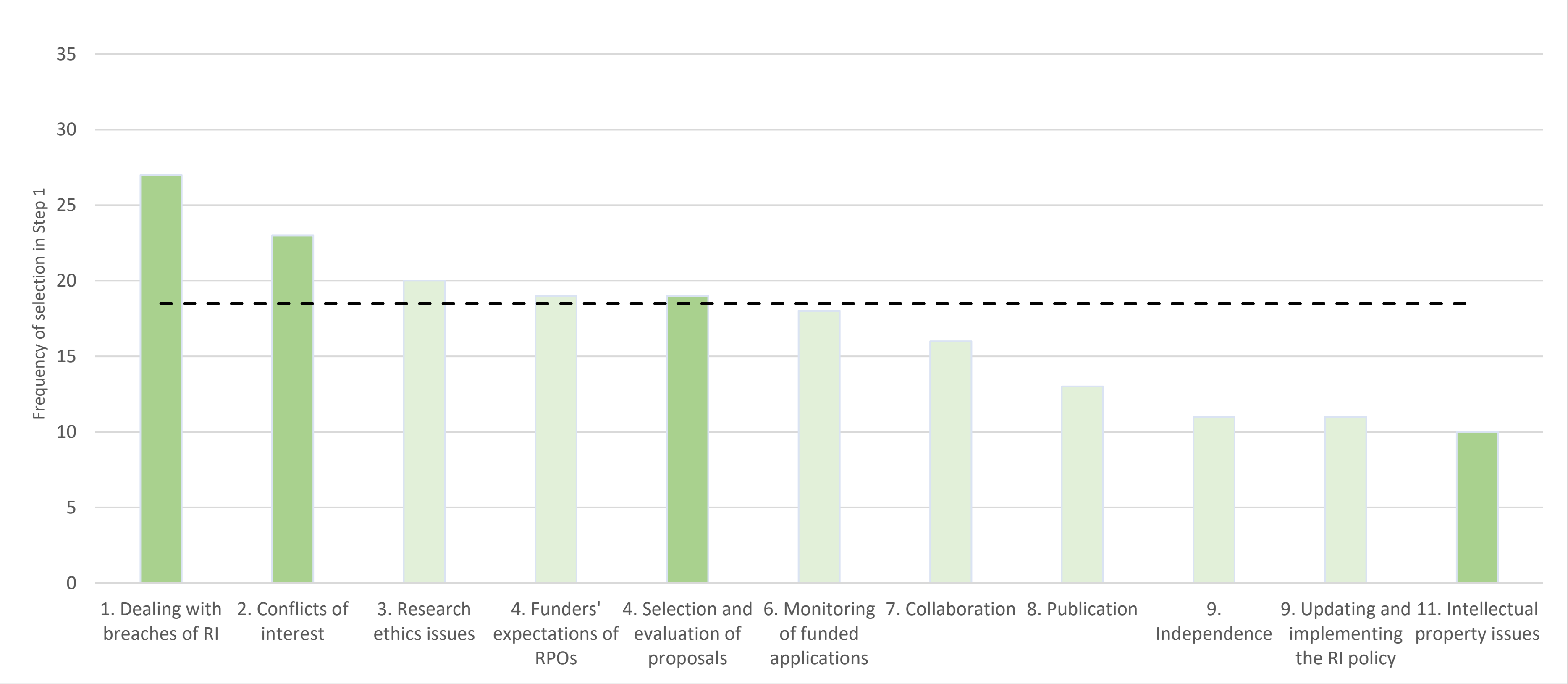

Figure 4 ***How often each topic was prioritized***. *37 experts took part in this exercise, so each topic could be selected a maximum of 37 times. The black dotted line represents 50% of respondents. The bars that are in dark green represent the topics that received the same ordering in Steps 1 and 2 of the prioritization and ranking exercise.*

Figure 5 shows the total ranking score of each topic.

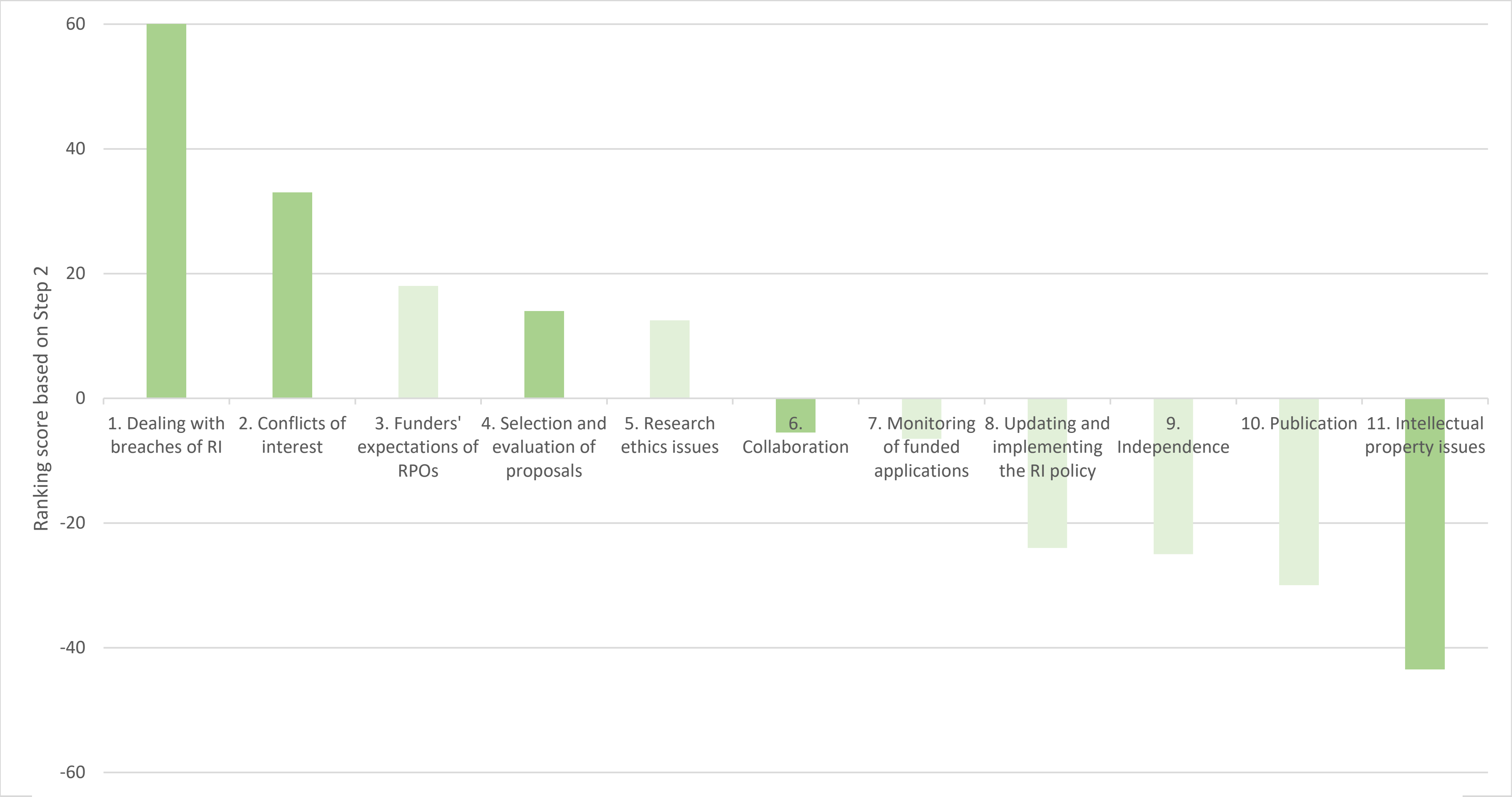

Figure 5 *The total ranking score of each topic. Topics with a higher ranking score received a higher ranking place. The bars that are in dark green represent the topics that received the same ranking place in Steps 1 and 2 of the ranking exercise.*

Table 2 shows an overview of the number of experts who were assigned each ranking score for each topic.

Table 2: Ranking scores per topic

**Topic**	**Rank**						**Total ranking score**
	**5**	**4**	**3**	**2**	**1**	**-2.5**	
**Dealing with breaches of RI**	6	8	6	1	5	11	62
**Conflicts of interest**	4	6	3	5	5	14	33
**Funders' expectations of RPOs**	5	5	2	5	2	18	18
**Selection and evaluation of proposals**	5	2	6	2	4	18	14
**Research ethics issues**	3	3	4	6	4	17	12.5
**Collaboration**	3	4	2	3	4	21	-5.5
**Monitoring of funded applications**	1	1	5	7	3	20	-6.5
**Updating and implementing the RI policy**	5	2	2	0	2	26	-24
**Independence**	4	3	2	0	2	26	-25
**Publication**	1	2	2	3	5	24	-30
**Intellectual property issues**	0	1	3	5	1	27	-43.5

This table represents the number of experts (text not in bold) who were assigned each ranking score per topic in Phase B of the analysis of the prioritization and ranking exercise.
