# Supplementary material for: Important Topics for Fostering Research Integrity by Research Performing and Research Funding Organizations: A Delphi Consensus Study
Source: Sci Eng Ethics. 2021 Jul 9;27(4):47. doi: 10.1007/s11948-021-00322-9 (PMC8270794; doi:10.1007/s11948-021-00322-9)
Supplement: Supplementary file 7 — Supplementary file7 (PDF 136 kb) [file 11948_2021_322_MOESM7_ESM.pdf]

Appendix 7: Ranked list of topics

Table 1: Ranked list of important RI topics for RPOs

Rank	Topic	Subtopics
1	Education and training in RI	a. pre-doctorate<br>b. post-doctorate<br>c. training of RI personnel & teachers<br>d. RI counselling and advice
2	Responsible supervision and mentoring	a. PhD guidelines<br>b. supervision requirements & guidelines<br>c. building and leading an effective team
3	Dealing with breaches of RI	a. RI bodies in the organisation<br>b. protection of whistleblowers<br>c. protection of those accused of misconduct<br>d. procedures for investigating allegations<br>e. sanctions<br>f. other actions
4	Supporting a responsible research process	a. research requirements<br>b. transparency<br>c. quality assurance
5	Data management	a. guidance and support<br>b. secure data storage infrastructure<br>c. FAIR principles
6	Research ethics issues	a. set-up and tasks of ethics committees<br>b. ethics review procedures
7	Conflicts of interest	a. in peer review<br>b. in the conduct of research<br>c. in appointments and promotions<br>d. in research evaluations<br>e. in consultancy
8	Research culture	a. fair procedures for appointments, promotions and remuneration<br>b. adequate education and skills training<br>c. culture building<br>d. managing competition & publication pressure<br>e. conflict management<br>f. diversity issues
9	Publication and communication	a. publication statement<br>b. authorship<br>c. open science<br>d. use of reporting guidelines<br>e. peer review<br>f. predatory publishing<br>g. communicating with the public
10	Updating and implementing the RI policy	*NONE*

11	Intellectual property issues	a. policies ensuring compliance with IP regulations<br>b. interaction of IP and open science requirements
12	Collaborative research among RPOs	a. among RPOs inside/outside the EU<br>b. with countries with different R&D infrastructures<br>c. between public and private RPOs

The rankings are based on the results of Round 2 of the Delphi study (the prioritization and ranking exercise). Topics ranked higher in priority have a lowe rank number (e.g. 'Education and training in RI' is the highest ranked topic). For a description of the topics, please see Online Resource 8. For a description of the subtopics, click here.

Table 2: Ranked list for important RI topics for RPOs

Rank	Topic	Subtopic
1	Dealing with breaches of RI	a. RI bodies in the organisation<br>b. by funded researchers<br>c. by review committee members<br>d. by reviewers<br>e. by staff members<br>f. protection of whistleblowers and the accused
2	Conflicts of interest	a. among review committee members<br>b. among reviewers<br>c. among staff members
3	Funders' expectations of RPOs	a. Codes of Conduct<br>b. assessment of researchers<br>c. education and training for RI<br>d. processes for investigating allegations of research misconduct
4	Selection & evaluation of proposals	a. RI plan<br>b. methodological requirements<br>c. plagiarism<br>d. diversity issues
5	Research ethics issues	a. research ethics requirements<br>b. ethics reporting requirements
6	Collaboration	a. expectations on collaborative research<br>b. research that is co-financed by multiple funders
7	Monitoring of funded applications	a. financial monitoring<br>b. monitoring of execution of research grant<br>c. monitoring of compliance with RI requirements
8	Updating and implementing the RI policy	*NONE*
9	Independence	a. What counts as an unjustifiable interference?<br>b. preventing unjustifiable interference by the funder<br>c. preventing unjustifiable interference by political or other external influences<br>d. preventing unjustifiable interference by commercial influences
10	Publication	a. publication requirements<br>b. expectations on authorship<br>c. open science
11	Intellectual property issues	*NONE*

The rankings are based on the results of Round 2 of the Delphi study (the prioritization and ranking exercise). Topics ranked higher in priority have a lowe rank number (e.g. 'Dealing with breaches of RI' is the highest ranked topic). For a description of the topics, please see Online Resource 8. For a description of the subtopics, click here.
