# Supplementary material for: Important Topics for Fostering Research Integrity by Research Performing and Research Funding Organizations: A Delphi Consensus Study
Source: Sci Eng Ethics. 2021 Jul 9;27(4):47. doi: 10.1007/s11948-021-00322-9 (PMC8270794; doi:10.1007/s11948-021-00322-9)
Supplement: Supplementary file 8 — Supplementary file8 (DOCX 244 kb) [file 11948_2021_322_MOESM8_ESM.pdf]

Appendix 8: Description of each topic

Table 1: Description of RPO topics to be included in a RIPP

Topic	Description	Types of policy
Education and training in RI	The RPO's plan on delivering successful RI trainings for different target groups including pre-doctorate researchers, post-doctorate researchers, and RI personnel & teachers. This topic also addresses the counseling and advice (online and offline) that the RPO will provide to employees to create awareness about RI.	- Training plans<br>- Infrastructure for counseling & advice (e.g. advisors, online information)
Responsible supervision and mentoring	The RPO's contain a policy on supervision and mentoring, including how to support PhD students in receiving good supervision, what requirements to set for supervisors and how to communicate them, as well as how to support research leaders in developing responsible research groups.	- Guidelines on supervision and mentoring<br>- Requirements that supervisors and team leaders should meet
Dealing with breaches of RI	How the RPO will address allegations of research misconduct. This includes which RI bodies will be installed to deal with RI breaches, how whistleblowers and accused researchers will be protected, what procedures for dealing with misconduct will be arranged, as well as the consequences that will follow from proven cases of misconduct.	- Procedures
Supporting a responsible research process	Things that RPOs will require from researchers to ensure that research is conducted responsibly from start to end (e.g. preregistering studies), as well as the services that the RPO will provide to ensure that research can be conducted transparently (e.g. institutional systems for reliable image processing). This topic also addresses the issue of how the RPO will monitor research for the purpose of quality assurance.	- Requirements<br>- Transparency infrastructures and systems<br>- Monitoring procedures

Research ethics issues	The organizational procedures and structures in place to ensure compliance with national and international research ethics requirements. This includes the RPO's policies on the set-up and tasks of ethics committees, as well as the procedures involved in ethics review.	- Research ethics bodies<br>- Procedures for ethics review
Data management	The support and tools that the RPO will provide to researchers to ensure good data management and compliance with the GDPR (e.g. secure data storage facilities).	- Data management tools and systems<br>- Requirements
Conflicts of interest	The institutional policy on what constitutes a conflict of interest and how that should be declared.	- Requirements
Research culture	The institutional policies in place to create a responsible research environment. This includes creating fair procedures for appointing, remunerating and promoting researchers, measures to deal with competition and publication pressure, culture building initiatives, ensuring the adequate education and skills training of researchers, ways to address conflicts, as well as policies for inclusivity and diversity.	- Procedures<br>- Initiatives
Publication and communication	The RPO's requirements on how researchers should publish and communicate their study results.	- Requirements
Updating and implementing the RI policy	The measures in place to ensure that the RPO successfully implements the RIPP and adequately updates it.	- Procedures
Intellectual property issues	Organizational policies on licensing, patents, and intellectual property issues.	- Requirements<br>- Infrastructure
Collaborative research among RPOs	RPOs' policies and procedures to ensure responsible collaboration with institutions inside/outside the EU, with countries with different R&D infrastructures, as well as private-public research collaborations. This includes issues such as benefit sharing	- Tools (e.g. data sharing agreements)<br>- Guidelines and requirements

	policies, data sharing agreements, etc.	

To see the evolution of the topic descriptions across the study, please consult the following links:

a) Topic descriptions before Round 1:

https://osf.io/jc6u2/?version=2&displayName=RPO%20list%20of%20topics-2019-05-10T11%3A09%3A19.833831%2B00%3A00.pdf

b) Topic descriptions before Round 2:

https://osf.io/jc6u2/?version=4&displayName=RPO%20list%20of%20topics-2019-08-21T14%3A57%3A47.568795%2B00%3A00.pdf

c) Final topic descriptions:

https://osf.io/jc6u2

Table 2: Description of RFO topics to be included in a RIPP

Topic	Description	Types of policy
Dealing with breaches of RI	How the RFO will address allegations of research misconduct related to funded researchers or reviewers and employees of the funder itself. This includes which RI bodies will be installed to deal with RI breaches, how whistleblowers and accused researchers will be protected, what procedures for dealing with misconduct will be arranged, as well as the consequences that will follow from proven cases of misconduct.	- Procedures
Conflicts of interest	The RFO's policy on what constitutes a conflict of interest and how that should be declared and dealt with responsibly.	- Requirements<br>- Procedures
Funders' expectations of RPOs	The RFO's policies and requirements of RPOs that receive funding. This includes requirements on following codes of conduct on RI, on assessing researchers, training researchers in RI, as well as dealing with breaches of RI.	- Requirements to RPOs<br>- Set up of communication channels with RPOs
Selection & evaluation of proposals	How the funder will ensure that it selects and evaluates research proposals responsibly to ensure that good research is awarded. This includes the RFO's requirements for proposals including the existence of an RI plan, establishing the need for the research, and methodological requirements. Additionally this topic also addresses how the funder will perform plagiarism checks and promote diversity and inclusivity in funded research.	- Procedures
Research ethics issues	The requirements the funder sets on research ethics issues, including research ethics requirements (e.g. obtaining ethics approval) and research ethics reporting requirements (e.g. mentioning ethics approval in proposals/publications).	- Requirements
Collaboration	The RFO's expectations on how to ensure that collaborations between institutions, or research funded by multiple funders, is done	- Requirements

	responsibly (e.g. requirements on data transfer agreements).	
Monitoring of funded applications	The funder's monitoring policy, including financial monitoring, monitoring of the execution of the research grant, and monitoring of compliance with RI standards.	- Procedures
Updating and implementing the RI policy	The measures in place to ensure that the RFO successfully implements the RIPP and adequately updates it.	- Procedures
Independence	The RFO's policy aimed at ensuring that research is not unjustifiably interfered with by the funder itself or by commercial, political or other external influences.	- Procedures
Publication	The RFO's requirements on how researchers should publish and communicate their study results.	- Requirements
Intellectual property issues	The RFO's policies on licensing, patents, and intellectual property issues.	- Requirements

To see the evolution of the topic descriptions across the study, please consult the following links:

a) Topic descriptions before Round 1:

https://osf.io/82dwk/?version=2&displayName=RFO%20list%20of%20topics-2019-05-10T11%3A09%3A08.195619%2B00%3A00.pdf

b) Topic descriptions before Round 2:

https://osf.io/82dwk/?version=4&displayName=RFO%20list%20of%20topics-2019-08-21T14%3A57%3A27.697726%2B00%3A00.pdf

c) Final topic descriptions:

https://osf.io/82dwk/
