# Supplementary material for: Important Topics for Fostering Research Integrity by Research Performing and Research Funding Organizations: A Delphi Consensus Study
Source: Sci Eng Ethics. 2021 Jul 9;27(4):47. doi: 10.1007/s11948-021-00322-9 (PMC8270794; doi:10.1007/s11948-021-00322-9)
Supplement: Supplementary file 9 — Supplementary file9 (PDF 214 kb) [file 11948_2021_322_MOESM9_ESM.pdf]

Appendix 9: Qualitative results per topic

Table 1: Summary of experts' comments for the RPO study (per topic)

Topic	Experts' views on the importance of the topic	Example quotes
**Education and training in RI**	• Problems with RI are often caused by lack of knowledge of RI. Increasing knowledge of RI among researchers will foster RI.<br>• Education is the key to prevention of RI breaches.	"The key to avoiding infringements of good research practice is training, often there might be honest error in play because the researchers don't know any better. We have to remediate this through training as much as possible." (Round 1)
**Responsible supervision and mentoring**	• In order to ensure that education and training for RI are effective, it is important to foster responsible supervision and mentoring in institutions<br>• Supervisors are role models for researchers.<br>• RI is not only the responsibility of the junior researchers, but also of the supervisors. Supervisors are responsible for educating students about RI.	"The supervisors and mentors are essential! Because they are role models." (Round 1)<br><br>"RI is also responsibility of supervisor; students do not always know how to deal with RI requirements" (Round 1)<br><br>"Mentoring and supervision to learn [RI] on-hands, if you educate and train and everybody still perform projects without RI will not help at all" (Round 3)
**Dealing with breaches of RI**	• Many institutions do not have a comprehensive approach to dealing with breaches of RI.<br>• Dealing with breaches of RI is complementary to a preventative approach to RI.<br>• Dealing with breaches of RI is an urgent issue for institutions.	"…many organisations - institues do not have a comprehenisve appraoch, they only have bits and pieces.. .but it needs to be a system..." (Round 1)<br><br>"[D]ealing with RI is one of the most important ways to prevent misconduct" (Round 1)<br><br>"…institutions need help with dealing with breaches right now" (Round 3)
**Research ethics issues**	• Research ethics is already an established topic for some disciplines, but there is a need for more awareness of this topic in non-biomedical disciplines.<br>• The topic is quite broad and not all aspects of research ethics can be captured in policies.	"…non medical areas are still unaware of its [research ethics'] importance" (Round 3)<br><br>"One should avoid 'ethics of science in general'[.] This is a very wide and multifacet[ed] world of issues. Cannot be covered [in RIPPs] satisfactorily" (Round 1)

	• The relationship between research ethics and research integrity is unclear	"question is do you keep ethics apart from integrity or not? how do they relate?"
**Data management**	• There is currently much ambiguity about how to comply with the GDPR and awareness of FAIR principles is low.<br>• Good data management is necessary to check the methodology of a study.<br>• Data management is an institutional, rather than researcher-only, responsibility.<br>• There are already many initiatives on improving research data management.	"Ambiguity over GDPR and low awareness of FAIR principles are the two principal reasons for this [high] rating." (Round 1)<br><br>"[Data management is the o]nly way to check proper methods and integrity" (Round 1)<br><br>"Data management is an is[sue] where a lot of responsibility is on institutional level - what systems they are able to provide." (Round 3)<br><br>"We have these policies in place." (Round 1)
**Supporting a responsible research process**	• This topic is important for conducting sound research.<br><br>• Institutions should have policies on this topic to help researchers. The basis for this topic is already available in institutions.	"…this issue is seen as essential in order to produce sound science - the main topic as well as subtopics all relate to basic research criteria for carrying out research projects (from design to c[ommu]nicating results)" (Round 3)<br><br>"These are practical things that need to be done, but are seen as administrative burden - so, everything that halps to ease this burden is important. I guess there is a practical hope from the researchers side that the institutions should provide more help in these more administrative matters to the researchers and take at least some of the burden off the shoulders of the researchers" (Round 3)
**Conflicts of interest**	• One of the major causes of breaches of RI are conflicts of interest<br>• Conflicts of interest are difficult to address and policies would be helpful.	"Conflict of interests is one of the major sources for breaching RI rules; should be identified and avoided at all costs" (Round 1)<br><br>"…we are overwhelmed by questions and requests for an opinion on this topic" (Round 3)

**Research culture**	• This is the basis for RI.<br>• Creating a good research culture is the ultimate goal of RI policies.<br>• The topic is related to other topics.<br>• There is already existing guidance on some of the subtopics within this topic, while others are underdeveloped.<br>• The topic is quite vague and difficult to address directly using institutional policies.	"we consider the research culture the main mean through w[h]ich we can ensure an effe[ct]ive RI within the institution" (Round 3)<br><br>" Other topics should be oriented to generate a culture change that will provide the organization a good research environment" (Round 3)<br><br>"Some of these [sub]topics (like appointments and promotions) are already covered by other procedural documents" (Round 1)<br><br>"Research culture …[is] too generic and therefore unmanageable for the majority of the experts." (Round 3)
**Publication and communication**	• RI problems and disagreements often deal with authorship disputes<br>• Open Science is important for RI<br>• This topic is already covered by many initiatives<br>• More policy on this topic will not lead to change.<br>• It is problems with the reward structure of science that lead to problems in publication and communication.	"Authorship disputes are common at my organisation and as a consequence authorshop dispute resolution is essential." (Round 1)<br><br>"[Open science] increases transparency, reproducibility, [and is] a must for publicly funded research" (Round 1)<br><br>"Maybe that respondents think that enough is known on this topic, yet, because of the personal issues, it is always difficult to deal with authorship issues?" (Round 3)<br><br>"The main issues relating RI and publication to both authorship and open science relate to the reward structures around current publication practices. It is the reward structures that need to change." (Round 3)
**Updating and implementing the RI policy**	• Implementation, monitoring and updating are crucial for a good RI policy<br>• This topic is not directly related to RI<br>• This topic is not directly relevant for research practice.	"RI policy is useless if it is not updated and implemented. (Round 3)<br><br>"[This topic is a] legal issue[] of no relevance for the people who manage RI in research institutions" (Round 3)

		“I guess these issues seem to situate outside of the standard of "doing researchers" and seem more fitting for leaders, managers, lawyers, PR-specialists and other supportive staff.” (Round 3)
**Intellectual property issues**	• This topic can also be dealt with by others (e.g. institutional technology transfer office).<br>• IP protection is necessary for innovation.<br>• This topic is very specific and legal.	“I think intellectual property issues are perhaps ranked quite low, as they are monitored through other channels, such as the legal department or a tech transfer office.” (Round 3)<br><br>I think the im[po]rtance of the topic is underestimated as there is more and more societaly pressure for different kinds of knowledge transfer and implementation of research results, where the IP issues become crucial. On the other hand - if you start to deal with IP, you drawn into legal jargon and nuances so the researchers better a[v]oid the topic in general.” (Round 3)
**Collaborative research among RPOs**	• Research is increasingly collaborative and there is a need for guidance on how to take part in responsible collaboration.<br>• It is important to come to agreements before the research starts in order to prevent problems of RI later.<br>• Before focusing on collaborations, it is first important to establish a good basis for RI within institutions<br>• Collaboration between researchers might not be effectively addressed via policy documents as it relates to interpersonal relationships.	“Incr[ea]sing part of research is collaborative; awareness and proper procedures needed” (Round 1)<br><br>“each institution is still consolidating its own RI procedures. Only at a later stage can you start to look outside of the institution.” (Round 3)<br><br>“…collaboration among RPOs, seems more of a formal topic that creates new collaboration possibilities but does not in itself cover the behaviour of individuals.” (Round 3)

Table 2: Summary of experts' comments for the RFO study (per topic)

Topic	Experts' views on the importance of the topic	Quotes
**Dealing with breaches of RI**	• Breaches of RI are highly damaging and urgent and should be dealt with well.<br>• Dealing with breaches of RI will have a high impact on researchers' behavior.<br>• Tackling this topic is complementary to a preventative approach to RI<br>• Dealing with breaches of RI is necessary to create a responsible research culture.	"…short-term there is the risk of reputation damage that requires immediate and effective action." (Round 3)<br><br>"real life shows that people start to take RI seriously very often only then if they understand the damage." (Round 3)<br><br>"For a funder, who is accountable for public funding it is vital to have assurance that breaches of good practice will be properly and fairly dealt with and not hidden or handled poorly or unfairly." (Round 3)<br><br>"…ways of dealing with RM create and maintain the culture of integrity of an institution" (Round 3)
**Conflicts of interest**	• Conflicts of interest have a high incidence, so it is necessary to deal with them appropriately.<br>• This topic is especially important in some disciplines (e.g. engineering)	"…conflicts of interest are always luring around. This is also in the process of selection and evaluation of proposals." (Round 3)<br><br>"All these CoI regularly happen and therefore it is important to be constantly aware of them, such that appropriate action can be taken" (Round 1)<br><br>"I would put "conflicts of interest" 1st, due to the fact that I come from the engineering/natural sciences field. There interaction with the industry is more straightforward with respect to Social Sciences and Humanities, where this topic might be of not such an importance." (Round 3)
**Research ethics issues**	• This topic is more important for some disciplines than others<br>• Unclarity exists about the relationship between research ethics and RI<br>• This topic is already well established	"Research ethics…is important but doesn't cut across all disciplines." (Round 3)<br><br>"[Research ethics] increases quality of research" (Round 1)

		“research ethics seem an important topic to me; in my understanding it does not belong to research integrity in the more narrow sense, though.” (Round 1)<br><br>“I come from a country where we have a well-established system of ensuring ethics issues in research are properly managed” (Round 1)
**Funders' expectations of RPOs**	• RPOs and RFOs each have a responsibility for RI. It is helpful to have a harmonization of policies.<br>• Funders have the power to set requirements on RPOs and researchers, and should take this role seriously<br>• Funders often influence researchers, indirectly through RPOs<br>• Funders’ expectations have a strong impact on research	“Research funding organisations have a lot of power (through funding) to influence policies as well as good practice.” (Round 3)<br><br>“Increasing dependence of rese[ar]ch performing organisations on external funding makes meeting the expectations of funders a priority.” (Round 3)
**Selection & evaluation of proposals**	• Research proposals should include a good motivation and a strong research and RI plan.<br>• Plagiarism is unacceptable and must be dealt with.	“Very important that plans for ensuring research integrity are included in the application” (Round 1)<br><br>“Any research proposal should contain a proper motivation and research plan. Plagiarism is absolutely unacceptable.” (Round 1)
**Monitoring of funded applications**	• Monitoring is important but difficult to do, regarding resources.<br>• Monitoring research project might not be feasible for RFOs	“[This topic is] very important, but unlikely to be externally monitorable without considerable oversight” (Round 1)<br><br>“…monitoring of ethics issue or realization of the project is problematic and not feasible for RFO to monitor. It requires time and experts..and money so it is better to transfer this issues on RPO.” (Round 3)
**Collaboration**	• It is helpful to have structures in place to deal with the topic.<br>• It is helpful to have a harmonization of policies across funders for projects funded by multiple funders	“People try to solve collaboration conflicts via an allegation of research misconduct. Knowing how to better deal with conflict[t]s could help avoid such allegations” (Round 1)<br><br>“Different funders may have different terms and conditions; can this work [i.e. research on RIPPs] help navigate that?” (Round 1)

**Publication**	• RFOs should prepare for the impact of Plan S on publication practices.<br>• This topic is already addressed by initiatives such as COPE<br>• The link between Open Science and RI is not clear<br>• Plan S is controversial<br>• This topic should be addressed by science editors and policy makers, instead of RFOs	"This is an area where many researchers are poorly informed, particularly around new developments such as Plan S." (Round 1)<br><br>"These are important issues, but may already be covered by organizaitons such as COPE." (Round 1)<br><br>"I don't understand this as Plan S goes too far as it demands that Open Access publications in hybrid journals are not permitted. In my field of science the most important journals are hybrid." (Round 3)<br><br>"Perhaps because publication is difficult to be dealt in the context of RPOs or RFOs but it needs the involvement of science editors and policy makers." (Round 3)
**Independence**	• Independence is essential for producing reliable research.<br>• Policy documents might not be able to address this topic effectively.<br>• There are no problems with this topic currently in Europe<br>• Independence is vague, but closely related to the concrete topic 'Conflicts of interest'	"It is important that research always keeps its independence from external factors, since otherwise the confidence in the results of the researchers will be seriously compromised."(Round 3)<br><br>"…institutes [i.e. RPOs] are independent and [] this independence is institutionalized by the state authorities." (Round 3)<br><br>""Independence" - a broad theme that can also be addressed in a way, when looking at COI [i.e. conflicts of interest]" (Round 3)<br><br>"…ensuring independence a ross a range of actors will be challenging…" (Round 3)
**Updating and implementing the RI policy**	• It is important to stay up to date with developments in research and RI<br>• This topic is not directly relevant for researchers	"This is an evolving policy space so staying abreast of current developments is important." (Round 1)

		"[This topic] is about organisational-administrative processes rather than about RI directly" (Round 3)
**Intellectual property issues**	• Intellectual property issues are legal issues and might be handled better elsewhere than in a RI promotion plan.<br>• This topic is already well established in some countries.<br>• This topics is more relevant for some disciplines than others.	"This question is unclear. In our jurisdiction, authorship disputes are within the scope of research integrity, but other intellectual property issues (e.g. patents) are considered legal issues." (Round 1)<br><br>"In my country, IP policies are well-defined at national level, with many supports in place." (Round 1)<br><br>"Again, coming from engineering/natural sciences field gives me a closer view of patenting and licencing issues that can become very important and sometimes detrimental for research." (Round 3)
