# Supplementary material for: Important Topics for Fostering Research Integrity by Research Performing and Research Funding Organizations: A Delphi Consensus Study
Source: Sci Eng Ethics. 2021 Jul 9;27(4):47. doi: 10.1007/s11948-021-00322-9 (PMC8270794; doi:10.1007/s11948-021-00322-9)
Supplement: Supplementary file 10 — Supplementary file10 (PDF 299 kb) [file 11948_2021_322_MOESM10_ESM.pdf]

Appendix 10: Experts' likely rationale for the prioritization and ranking exercise (Results of Round 3 of the Delphi study)

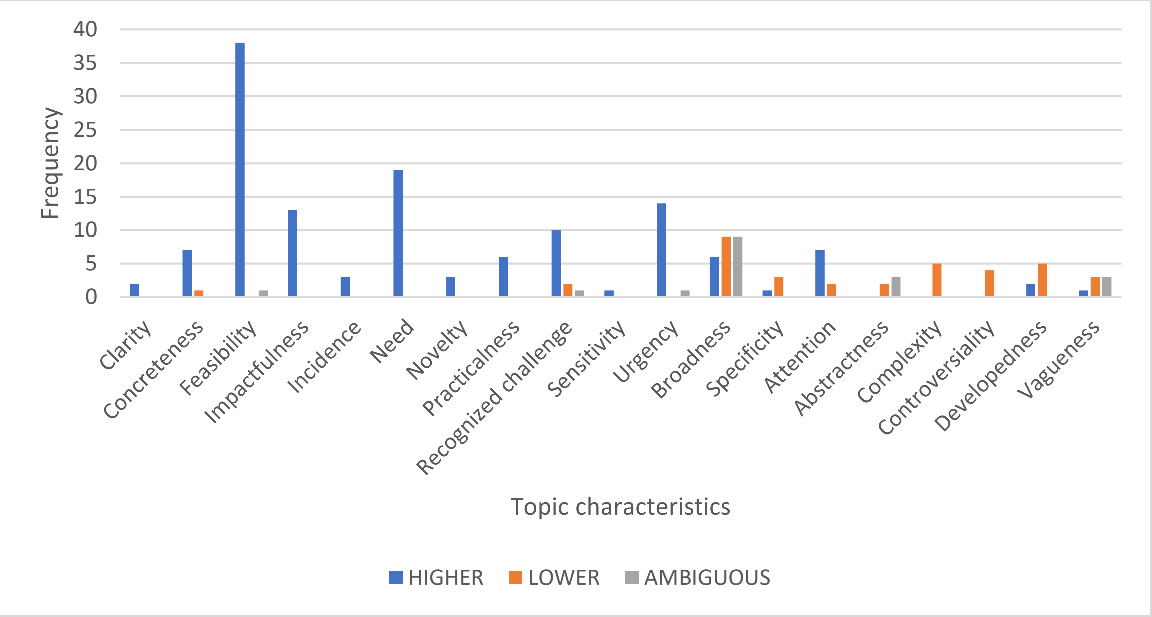

Figure 1 Factors that likely influenced experts' ranking

The factors mentioned are derived from results of Round 3 of the Delphi study. The x-axis represents the topic characteristics that experts in Round 3 mentioned influenced the rankings. The y-axis represents the number of times that experts in Round 3 identified the topic characteristic as influencing the topic to be ranked higher (blue), lower (orange), or in an ambiguous way (i.e. influenced some experts to rate the topic higher and others low; in gray).

Evaluation of ranking results

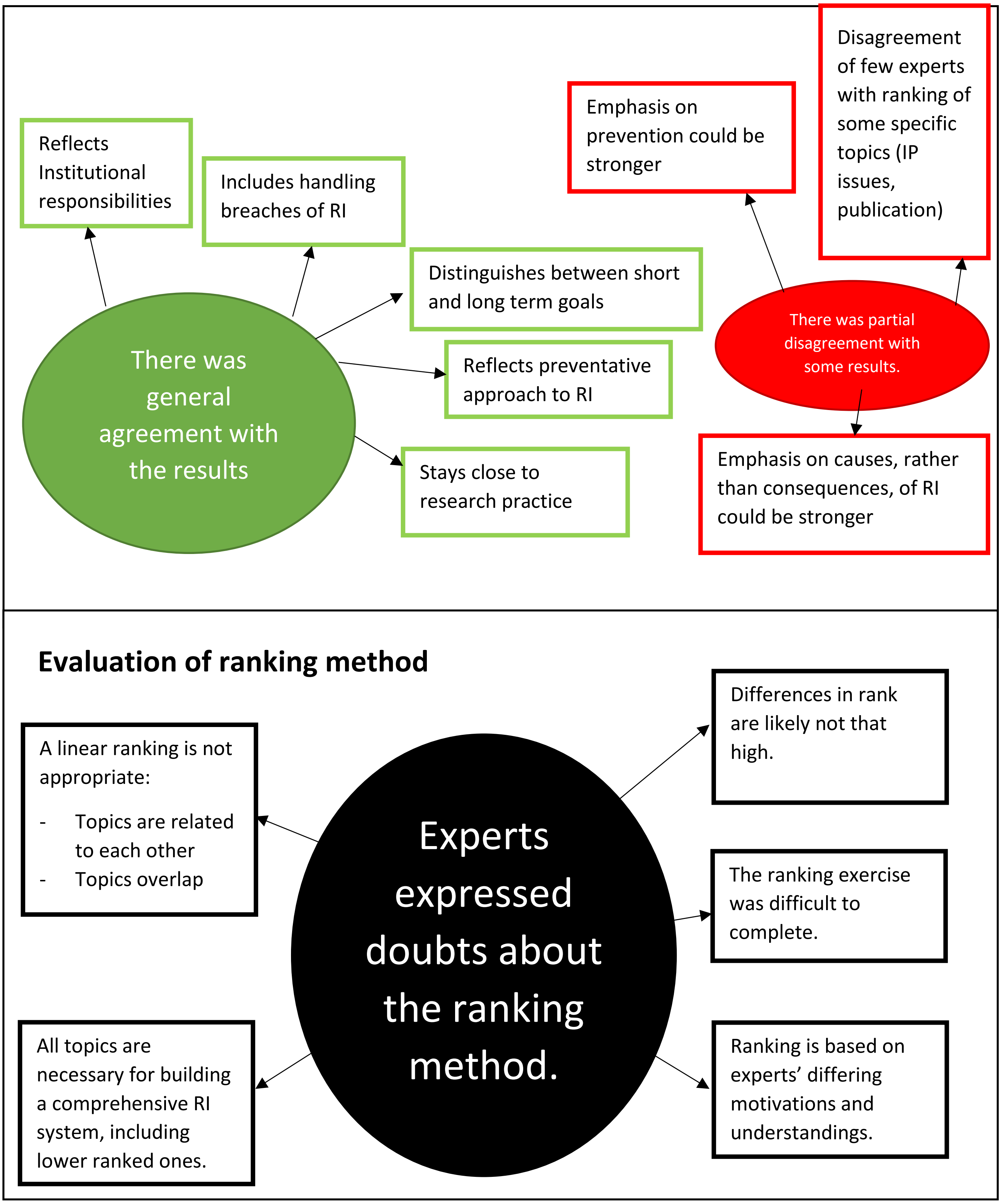

Figure 2 Experts' evaluations of the ranking. *This figure provides a summary of experts' views on the results of the ranking exercise (top box) and the ranking exercise method itself (bottom box).*
