# Supplementary material for: Important Topics for Fostering Research Integrity by Research Performing and Research Funding Organizations: A Delphi Consensus Study
Source: Sci Eng Ethics. 2021 Jul 9;27(4):47. doi: 10.1007/s11948-021-00322-9 (PMC8270794; doi:10.1007/s11948-021-00322-9)
Supplement: Supplementary file 11 — Supplementary file11 (PDF 693 kb) [file 11948_2021_322_MOESM11_ESM.pdf]

Appendix 11: Ethics approval

J. Tijdink MD, PhD
Afdeling Metamedica
F-vleugel F-033

Medisch Ethische Toetsingscommissie VUmc
Van der Boechorststraat 7, kamer H-443
Postbus 7057
1007 MB Amsterdam
020 444 5585
www.vumc.nl/METc

Datum:	21 maart 2019
Ons kenmerk:	2019.152
Betreft:	Niet-WMO advies

Geachte heer Tijdink,

Het Dagelijks Bestuur van de Medisch Ethische Toetsingscommissie VU medisch centrum heeft uw onderzoek **Topics for standard operating procedures and guidelines on research integrity; a Delphi survey** besproken in de vergadering van 19/03/2019.

Het onderzoek valt niet onder de reikwijdte van de Wet Medisch-wetenschappelijk Onderzoek met mensen (WMO).

Het oordeel is gebaseerd op de volgende documenten:

Sectie	Onderwerp	Versie
A1	aanbiedingsbrief	d.d. 28-2-2019
C1	onderzoeksprotocol	versie 1 d.d. 28-2-2019
E11	informatiebrief	versie 1 d.d. 28-2-2018
E2	toestemmingsverklaring	versie 1 d.d. 28-2-2018
F1	vragenlijst	Delphi questionnaire

Het Dagelijks Bestuur van de Medisch Ethische Toetsingscommissie VU medisch centrum wijst u erop dat hoewel het ingediende onderzoek niet onder de reikwijdte van de WMO valt, andere wet- en regelgeving (mogelijk) wel van toepassing is, waaronder:

- WGBO (Wet Geneeskundige BehandelingsOvereenkomst);
- AVG (Algemene Verordening Gegevensbescherming), zie https://autoriteitpersoonsgegevens.nl/nl/onderwerpen/avg-europese-privacywetgeving;
- Code Goed Gedrag (Gedragscode gezondheidsonderzoek: gebruik medische gegevens in wetenschappelijk onderzoek), zie www.federa.org;

- Code Goed Gebruik (Gedragscode Verantwoord omgaan met lichaamsmateriaal ten behoeve van wetenschappelijk onderzoek, 2011), zie www.federa.org;
- Biobanken: Reglement toetsing biobank VUmc, zie https://www.vumc.nl/afdelingen/METc/biobank/;
- WBO (Wet Bevolkings Onderzoek), zie http://www.vumc.nl/afdelingen/METc/wetgeving/wetbevolkingsonderzoek/.

To whom it may concern

We are pleased to confirm that the Medical Research Involving Human Subjects Act (WMO) does not apply to the above mentioned study and that an official approval of this study by our committee is not required.

The Medical Ethics Review Committee of VU University Medical Center is registered with the US Office for Human Research Protections (OHRP) as IRB00002991. The FWA number assigned to VU University Medical Center is FWA00017598.

Met vriendelijke groet,
namens de Medisch Ethische Toetsingscommissie VU medisch centrum,

i/o

prof. dr. J.A.M. van der Post, voorzitter

c.c.: afdelingshoofd afdeling Metamedica prof.dr. G.A.M. Widdershoven
c.c.: k.labib@vumc.nl

Geachte | Dear Ms. Labib,

-Dutch-

De Vaste Commissie Wetenschap en Ethiek van de Faculteit der Gedrags- en Bewegingswetenschappen(VCWE), Vrije Universiteit Amsterdam, heeft de ethische aspecten van het door u ingediende onderzoeksvoorstel met als titel "Topics for standard operating procedures and guidelines on research integrity; the Delphi survey" beoordeeld.

Op basis van de voorgelegde informatie verklaart de VCWE dat het onderzoeksvoorstel voldoet aan de ethische richtlijnen van de faculteit. Dit positieve advies geldt voor een periode van 5 jaar na dagtekening.

Wij wensen u veel succes met uw onderzoek.

-English-

The Scientific and Ethical Review Board (VCWE) of the Faculty of Behavior & Movement Sciences, VU University Amsterdam, has reviewed your research proposal entitled "Topics for standard operating procedures and guidelines on research integrity; the Delphi survey" on ethical aspects.

Based on the submitted information, the board declares that the research proposal complies with the ethical guidelines of the faculty. This positive advice is valid for 5 years after today's date.

We wish you all the best in your research.

Met vriendelijke groet | With kind regards,

Dr. Annet Kleiboer
Chair of VCWE
